# Transcriptome profiles in peripheral white blood cells at the time of artificial insemination discriminate beef heifers with different fertility potential

**DOI:** 10.1186/s12864-018-4505-4

**Published:** 2018-02-09

**Authors:** Sarah E. Dickinson, Brock A. Griffin, Michelle F. Elmore, Lisa Kriese-Anderson, Joshua B. Elmore, Paul W. Dyce, Soren P. Rodning, Fernando H. Biase

**Affiliations:** 1 Department of Animal Sciences, Auburn University, 559 Devall Dr, Auburn, AL 36839 USA; 2 Alabama Cooperative Extension System, Auburn, AL USA

**Keywords:** Biomarkers, Infertility, Pregnancy

## Abstract

**Background:**

Infertility is a longstanding limitation in livestock production with important economic impact for the cattle industry. Female reproductive traits are polygenic and lowly heritable in nature, thus selection for fertility is challenging. Beef cattle operations leverage estrous synchronization in combination with artificial insemination (AI) to breed heifers and benefit from an early and uniform calving season. A couple of weeks following AI, heifers are exposed to bulls for an opportunity to become pregnant by natural breeding (NB), but they may also not become pregnant during this time period. Focusing on beef heifers, in their first breeding season, we hypothesized that: a- at the time of AI, the transcriptome of peripheral white blood cells (PWBC) differs between heifers that become pregnant to AI and heifers that become pregnant late in the breeding season by NB or do not become pregnant during the breeding season; and b- the ratio of transcript abundance between genes in PWBC classifies heifers according to pregnancy by AI, NB, or failure to become pregnant.

**Results:**

We generated RNA-sequencing data from 23 heifers from two locations (A: six AI-pregnant and five NB-pregnant; and B: six AI-pregnant and six non-pregnant). After filtering out lowly expressed genes, we quantified transcript abundance for 12,538 genes. The comparison of gene expression levels between AI-pregnant and NB-pregnant heifers yielded 18 differentially expressed genes (DEGs) (*ADAM20*, *ALDH5A1*, *ANG*, *BOLA-DQB*, *DMBT1*, *FCER1A*, *GSTM3*, *KIR3DL1*, LOC107131247, LOC618633, *LYZ*, *MNS1*, *P2RY12*, *PPP1R1B*, *SIGLEC14*, *TPPP*, *TTLL1*, *UGT8*, eFDR≤0.02). The comparison of gene expression levels between AI-pregnant and non-pregnant heifers yielded six DEGs (*ALAS2*, *CNKSR3*, *LOC522763*, *SAXO2*, *TAC3*, *TFF2*, eFDR≤0.05). We calculated the ratio of expression levels between all gene pairs and assessed their potential to classify samples according to experimental groups. Considering all samples, relative expression from two gene pairs correctly classified 10 out of 12 AI-pregnant heifers (*P* = 0.0028) separately from the other 11 heifers (NB-pregnant, or non-pregnant).

**Conclusion:**

The transcriptome profile in PWBC, at the time of AI, is associated with the fertility potential of beef heifers. Transcript levels of specific genes may be further explored as potential classifiers, and thus selection tools, of heifer fertility.

**Electronic supplementary material:**

The online version of this article (10.1186/s12864-018-4505-4) contains supplementary material, which is available to authorized users.

## Background

Female infertility remains a limiting factor in cattle production systems. In beef heifers, pregnancy rates vary from 53% to 95% [1–10] under natural breeding (NB), and are reduced to the range of 48–69% [1, 4, 7, 9, 11, 12] if artificial insemination (AI) is the only breeding strategy utilized. Best management practices in heifer development have been used to increase the probability of reproductive success in a heifer’s first breeding season [13]. For instance, heifers that reach 60% of their mature body weight [10], have a body conformation compatible with a healthy and well-nourished animal [3, 14], present reproductive structures indicative of cyclic animals [9, 15, 16], and are bred on their third estrus versus earlier cycles [8] may have a greater chance of becoming pregnant early in the breeding season [13]. Yet, under appropriate management, many of the heifers that are deemed reproductively mature according to morphological assessment and age criteria do not become pregnant. Unexplained infertility of otherwise healthy females impacts the cattle industry negatively and is a condition of significant importance in other livestock and humans [17].

In addition to the economic losses from infertile animals, heifers that conceive late in their first breeding season to NB are likely to cause losses to beef cattle operations. Following an unsuccessful AI, heifers that become pregnant to NB and calve after the first 21 days into their first calving season remain productive in the herd for a shorter period of time and wean less total pounds of calf than their early calving counterparts [18]. Therefore, improving the selection for heifers that become pregnant by AI at the beginning of the breeding season will reduce economic losses in beef cattle operations.

Genetic selection has been used extensively to improve production and reproductive traits in beef cattle operations. In heifers, fertility is assessed by first service conception and pregnancy rate. Nonetheless, low heritability estimates for pregnancy rate (0.07–0.13 [1, 4, 19]) and first service conception (0.02–0.18 [1, 4, 19, 20]) make it challenging to leverage statistical models to guide the decision making process for sire selection to improve female fertility in cattle. As a consequence, selection for fertility in beef heifers using traditional approaches has not achieved significant progress over generations.

Strategies leveraging molecular genetics biotechnology have added new perspective to understanding the genetic architecture of fertility. To that end, genomic polymorphisms [20–24], differential gene transcription in the hypothalamus [20], endometrium [25–29], and metabolites from follicular fluids [30] have been associated with fertility in heifers or cows. In women, investigation of circulating prognostic biomarkers have yielded promising candidates that are predictive of infertility [31], in vitro fertilization [32], or pregnancy outcomes [32, 33]. These studies, and the physiological connection between reproduction and the immune system [34], support the rationale that peripheral white blood cells (PWBC) harbor invaluable molecular information predictive of the physiological state of beef heifers pertaining to their likelihood of pregnancy establishment.

The molecular profile of circulating miRNAs [35] in the bloodstream and gene expression of PWBC [36] change during the early stages of pregnancy. Nonetheless, the molecular profiles of gene or protein expression in PWBC prior to fertilization have not been investigated as biomarkers for fertility in cattle. In this study, we tested the hypotheses that at the time of AI in beef heifers on their first breeding season: a- the transcriptome of PWBC differs between heifers that become pregnant to AI and heifers that become pregnant late in the breeding season by NB or do not become pregnant during the breeding season; and b- the ratio of transcript abundance between genes in PWBC classifies heifers according to pregnancy by AI, NB, or failure to become pregnant.

## Results

### Experiment overview

**Fig. 1:**
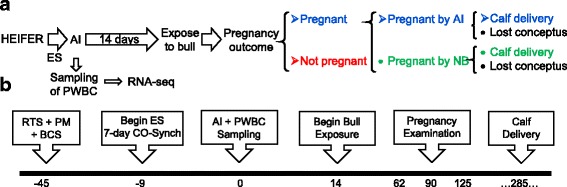
Overview of the experimental design and heifer classification. **a** General scheme used for the classification of heifers. **b** Depiction of the timeline adopted from breeding soundness evaluation to final heifer classification. See text for details. ES: estrous synchronization; AI: artificial insemination; RTS: reproductive tract scores; PM: pelvic measurements; BCS: body condition score

The experimental scheme of this study is outlined in Fig. 1a. Sixty pubertal, crossbred heifers (Angus x Simmental) were subjected to estrous synchronization followed by fixed-time AI with semen of proven fertility at two Auburn University Alabama Agricultural Experiment Stations. The heifers were then exposed to bulls for natural breeding and checked for pregnancy by rectal palpation. Figure 1b depicts the timeline of the experiment from breeding soundness to heifer classification. Heifers were identified as pregnant or not pregnant, and conceptus morphology was used to identify when conception occurred over the breeding season, for the classification of three groups (AI-pregnant, NB-pregnant, non-pregnant, Fig. 1a). We selected heifers from two experimental stations for transcriptome analyses. From station A, we carried out the experiment with AI-pregnant (*N* = 6) and NB-pregnant (*N* = 5) heifers. From station B, we conducted the experiment with AI-pregnant (*N* = 6) and non-pregnant (*N* = 6). Heifers presented similar averages for age, weaning weight, pelvic metrics, body condition score, and reproductive tract score within stations (*P* > 0.1, Additional file 1: Table S1).

For each heifer, we collected peripheral blood at the time of AI, and assayed high throughput sequencing from PWBC. We generated over 557.2 million pairs of reads, averaging 20.9 million pairs of reads uniquely aligned to the bovine genome UMD3.1 [37] per sample (Additional file 1: Table S2).

### Gene expression levels in PWBC associated with pregnancy outcome

**Fig. 2:**
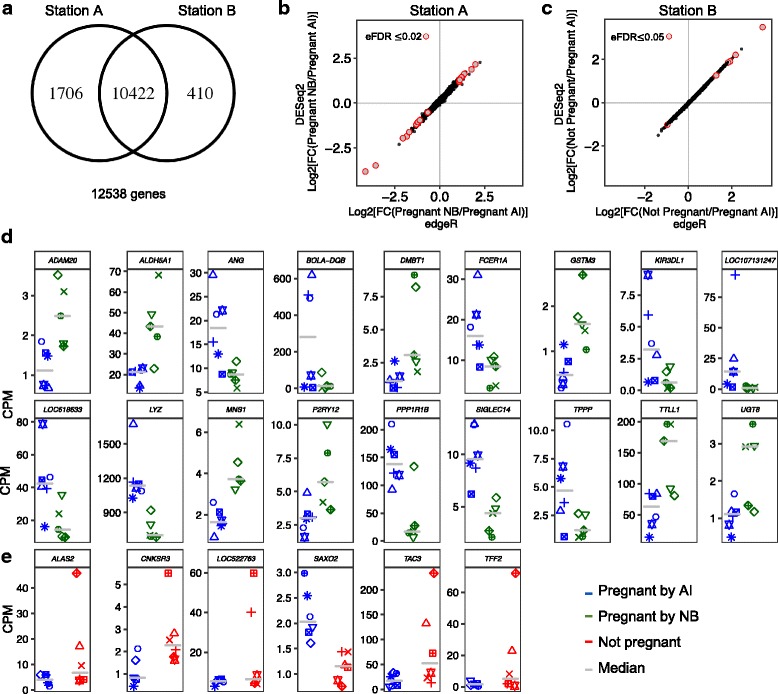
Gene expression levels associated with pregnancy outcome. **a** Number of genes with expression estimated in PWBCs. **b**, **c** Fold change profiles obtained by two Bioconductor packages highlighting the genes inferred as differentially expressed between the two experimental groups. **d**, **e** Expression levels (counts per million, CPM) for the DEGs obtained in experimental station A (**d**) and B (**e**). Within location, the shapes represent the same animals across gene charts

We counted pairs of reads [38] according to the bovine Ensembl annotation [39] to estimate transcript abundance of expressed genes. In order to remove quantification uncertainty associated to lowly expressed genes and erroneous identification of differentially expressed genes [40, 41], we retained genes with more than one count per million (1 CPM) in six or more samples for downstream analyses, for each location independently. We quantified expression levels of 12,538 genes in all samples. Of these genes, 10,422 were expressed in PWBC of heifers located at both experimental stations. Furthermore, 1706 and 410 genes were exclusively expressed in PWBC of heifers located at experimental stations A or B, respectively (Fig. 2a). In order to strengthen the inferences of differentially expressed genes (DEG) between heifers of differential pregnancy classification, we analyzed the data from each station independently, and we adopted two algorithms implemented in the Bioconductor [42] packages edgeR [43] and DESeq2 [44]. The fold changes estimated by both algorithms were very similar (r > 0.99, *p* < 0.0001) and we used the output from edgeR package to report the fold changes of differential gene expression.

The comparison of gene expression profiles in PWBC between AI-pregnant and NB-pregnant heifers resulted in 18 DEGs (Fig. 2b, eFDR≤0.02, Additional file 1: Figure S1), of which *DMBT1*, *ADAM20*, *ALDH5A1*, *GSTM3*, *MNS1*, *P2RY12*, *TTLL1*, *UGT8* showed greater and *ANG*, *BOLA*-*DQB*, *FCER1A*, *KIR3DL1*, LOC107131247, LOC618633, *LYZ*, *PPP1R1B*, *SIGLEC14*, *TPPP* displayed lower expression levels in NB-pregnant compared to AI-pregnant heifers (Table 1, Fig. 2d). Despite the low number of DEGs, we identified significant enrichment (FDR≤0.002) for the GO biological process “metabolic process” (*ALDH5A1*, *GSTM3, LYZ, UGT8*).

**Table 1:** Differentially expressed genes associated with pregnancy originated from artificial insemination or natural breeding

Ensembl ID	Symbol	Description	LogFC(pregnant NB/pregnant AI)^b^
ENSBTAG00000022715	*DMBT1* ^a^	Deleted in Malignant Brain Tumors 1	1.98
ENSBTAG00000001842	*GSTM3*	glutathione S-transferase Mu 3	1.76
ENSBTAG00000012030	*TTLL1*	tubulin tyrosine ligase like 1	1.36
ENSBTAG00000000271	*MNS1*	meiosis specific nuclear structural 1	1.28
ENSBTAG00000021902	*ALDH5A1*	aldehyde dehydrogenase 5 family member A1	1.20
ENSBTAG00000004574	*UGT8*	UDP glycosyltransferase 8	1.13
ENSBTAG00000038377	*ADAM20*	ADAM metallopeptidase domain 20	1.11
ENSBTAG00000015837	*P2RY12*	purinergic receptor P2Y12	1.08
ENSBTAG00000026779	*LYZ*	Lysozyme C, non-stomach isozyme	−0.67
ENSBTAG00000045492	*ANG* ^a^	angiogenin, ribonuclease, RNase A family, 5	−1.11
ENSBTAG00000040580	LOC618633^a^	myeloid-associated differentiation marker-like	−1.22
ENSBTAG00000012887	*FCER1A*	Fc fragment of IgE receptor Ia	−1.28
ENSBTAG00000035868	*SIGLEC14* ^a^	Sialic Acid Binding Ig Like Lectin 14	−1.31
ENSBTAG00000047116	*TPPP*	tubulin polymerization promoting protein	−1.70
ENSBTAG00000006035	*PPP1R1B*	protein phosphatase 1 regulatory inhibitor subunit 1B	−1.84
ENSBTAG00000047971	*KIR3DL1*	killer cell immunoglobulin-like receptor, three domains, long cytoplasmic tail, 1 precursor	−2.05
ENSBTAG00000021077	*BOLA-DQB* ^a^	major histocompatibility complex, class II, DQ beta	−3.57
ENSBTAG00000047764	LOC107131247	multidrug resistance-associated protein 4-like	−4.15

^a^Genes annotated manually according to either Keeg pathways, Uniprot or NCBI Entrez databases

^b^LogFC: log fold change output by edgeR package

The comparison of gene expression profiles in PWBC between AI-pregnant and non-pregnant heifers resulted in six DEGs (eFDR≤0.05, Fig. 2c, Additional file 1: Figure S1). The genes *ALAS2*, *CNKSR3*, LOC522763, *TAC3*, *TFF2* presented greater transcript abundance in non-pregnant heifers, whereas transcripts for *SAXO2* were less abundant in PWBC of non-pregnant heifers compared to heifers that became pregnant to AI (Table 2, Fig. 2e). No significant GO term was identified when these six DEGs where tested for enrichment of biological processes or molecular functions.

**Table 2:** Differentially expressed genes associated with pregnancy outcome in beef heifers

Ensembl ID	Gene symbol	Description	LogFC(not pregnant/pregnant AI)^b^
ENSBTAG00000030814	*TFF2*	trefoil factor 2	3.42
ENSBTAG00000021807	*TAC3*	tachykinin 3	2.17
ENSBTAG00000001308	LOC522763^a^		1.92
ENSBTAG00000013178	*ALAS2*	5′-aminolevulinate synthase 2	1.82
ENSBTAG00000012674	*CNKSR3*	CNKSR family member 3	1.28
ENSBTAG00000003414	*SAXO2*	stabilizer of axonemal microtubules 2	−0.98

^a^Genes annotated manually according to NCBI Entrez databases

^b^LogFC: log fold change output by edgeR package

We selected the genes *ALDH5A1*, *FCER1A*, LOC522763, *SIGLEC14*, *TAC3,* and *TTLL1* for independent assessment of differential gene expression by quantitative real-time polymerase chain reaction (qPCR). The averages of fold change calculated from the PCR data were correspondent to those obtained from RNA-seq (Spearman’s correlation = 0.94, *P* < 0.02, Additional file 1: Table S3). Therefore, we validated the results obtained by RNA-seq.

### Detection of gene pairs to discriminate heifers pregnant by AI

Next, we used the top scoring pair (TSP) approach [45] to test whether the ratio between transcript levels of two genes within samples discriminated heifers presenting distinct pregnancy outcomes. According to this approach, a gene’s expression level is compared to the expression levels of all other genes. For instance, in station A, 12,128 genes formed 147,076,256 pairs, and 10,422 genes in station B formed 117,321,392 pairs.

**Fig. 3:**
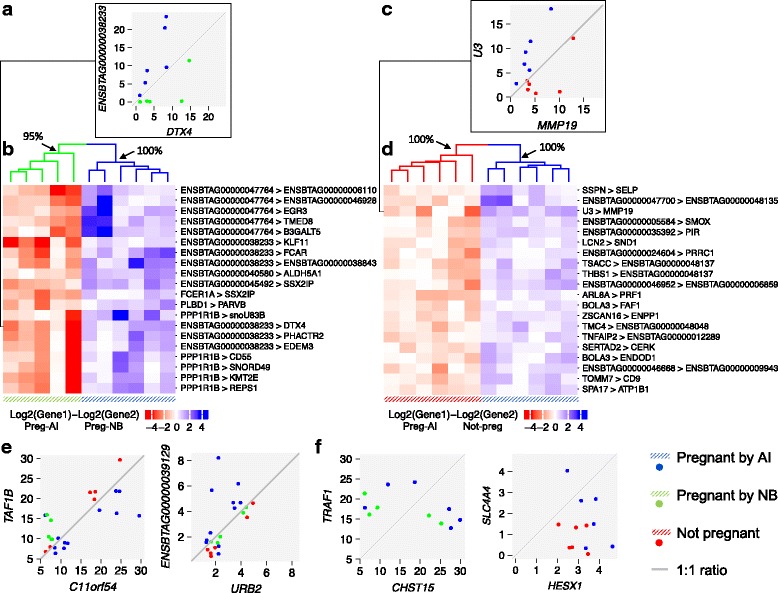
Top scoring pairs for sample classification. Top 20 gene pairs whose expression ratios separate the samples into two groups: AI-pregnant relative to NB-pregnant in station A (**a**, **b**) and AI-pregnant relative to non-pregnant in station B (**c**, **d**). **e** Identification of two TSP with significant separation of AI-pregnant heifers from the others (NB-pregnant, non-pregnant). **f** Pairs of genes randomly chosen to demonstrate the null hypothesis of the top scoring pair approach

The analysis of the transcriptome data from AI-pregnant and NB-pregnant heifers (station A) resulted in 1520 pairs of genes that discriminate most of the heifers according to their pregnancy outcome (Overall score = 1, *P* < 0.0002, 5000 randomizations). The pair of genes with the greatest discriminatory score was *DTX4* and ENSBTAG00000038233, whereby the transcript levels of *DTX4* are greater than the transcript levels of ENSBTAG00000038233 in NB-pregnant in contrast with AI-pregnant heifers (Fig. 3a). Clustering of the samples using the ratios of transcript levels of the top 20 gene pairs (Additional file 1: Figure S2a) separated the heifers into two clusters that followed their pregnancy classification (Fig. 3b, *P*≤0.01, 5000 randomizations).

Analysis of the transcriptome data from 12 heifers sampled from station B, (AI-pregnant and non-pregnant) resulted in 88 gene pairs identified that separated most of the heifers in two groups (Overall score = 1, P < 0.0002, 5000 randomizations). The genes *U3* and *MMP19* formed the top scoring pair, in which the AI-pregnant heifers presented greater transcript abundance of *U3* compared to *MMP19*, and the opposite direction was observed for the non-pregnant heifers (Fig. 3c). Clustering of the samples using the ratios of transcript levels of the top 20 gene pairs (Additional file 1: Figure S2b) resulted in the formation of two clusters that separated the samples by pregnancy outcome (Fig. 3d, *P*< 0.01, 5000 randomizations).

The TSP approach uses within subject transcript levels to calculate ratios between genes, and the analysis does not use variables that may create batch effects in animal experiments (i.e. time, genetic background, location of sampling). Thus, we interrogated the entire dataset (23 samples) under the binary classification of AI-pregnant (*N* = 12) and AI-not-pregnant (*N* = 11). There were four genes forming two pairs (*C11orf54*, *TAF1B*; *URB2*, ENSTAG00000039129) that discriminated 10 out of 12 heifers correctly (Fig. 3e, Overall score = 0.83). The clustering of 10 out of 12 AI-pregnant heifers independently from NB-pregnant and non-pregnant heifers, showed non-trivial (*P* < 0.003, hypergeometric test) patterns of ratios that identified heifers by pregnancy outcome, and clearly contrasted with ratio patterns obtained from random gene pairs (Fig. 3f).

## Discussion

Our main goal was to identify differences in the transcriptome profile in PWBC at the time of AI in beef heifers with different pregnancy outcomes. In our experiment, we identified heifers that became pregnant to AI, to natural breeding, and heifers that failed to become pregnant during the breeding season. Sampling blood from heifers of similar age and other phenotypic parameters within herd was central for us to work with pubertal heifers of similar nutritional status, and thus focus on the differences associated with the physiology of the reproduction driving the likelihood of pregnancy in beef heifers. Similar to other models of fertility and infertility in cattle [25–28], the categorical pregnancy outcomes adopted in our study identify heifers with distinct fertility potential. In the current study, we identified that variations in gene expression profiles of PWBC may be associated with the likelihood of a successful fertilization and pregnancy establishment.

Considering the similarity of the heifers within location, as observed by age, phenotypic records (Additional file 1: Table S1), genetic background, reproductive, health, and nutritional management, and other environmental effects within station, one could anticipate the low number of DEGs inferred in this study. Our very stringent approach for inferring DEGs according to two independent algorithms was one reason for this observation. Nonetheless, this strategy [46] greatly reduces the chance of inferring false positives by leveraging the strengths of both algorithms [47]. Other transcriptome investigations of endometrial tissues of beef heifers [27–29] or dairy cows [48] of different fertility potential yielded DEGs in the order of few dozens. Of note, no previously identified DEGs have been found in more than one study. Furthermore, none of the DEGs identified in our study were observed in similar investigations focusing on women’s fertility [31–33]. This observation is not surprising given the polygenic and complex physiology involving fertilization and pregnancy in females.

We identified 18 DEGs associated with heifer pregnancy to AI compared to pregnancy from natural breeding. Gene ontology analysis showed significant enrichment of the biological process “metabolic process”, which included the genes “aldehyde dehydrogenase 5 family member A1” (*ALDH5A1*), “glutathione S-transferase Mu 3” (*GSTM3*), “UDP glycosyltransferase 8” (*UGT8*), and “Lysozyme C, non-stomach isozyme” (*LYZ*). The gene *ALDH5A1* is part of a family of aldehyde dehydrogenases that metabolizes aldehydes and reduces cellular toxicity. Additionally, there is evidence, in humans, that a functional ALDH5A1 is associated with the concentration of glutathione in the bloodstream [49]. Also in humans, it has been hypothesized that upregulation of *GSTM3* is a response to greater presence of cytotoxic products resultant of overabundance of reactive oxygen species (ROS) [50] and the need for the conjugation of ROS to glutathione [51] to mitigate the toxic effects of ROS. As evidence supports the link between oxidative stress and female infertility in humans [51–53], the upregulation of *ALDH5A1* and *GSTM3* in PWBC suggests that a greater presence of ROS species in the blood stream may reduce the likelihood of pregnancy success to AI in beef heifers, but do not prevent the heifers from becoming pregnant to a bull later in the breeding season.

Although no significant enrichment was observed, it was noteworthy that four out of 18 DEGs were related to “cytoskeleton organization” (*MNS1, TTLL1*, *TPPP*, *UGT8*). Interestingly, *UGT8* was down-regulated in the endometrium of women affected by implantation failure [54]. The gene *FCER1A* is associated with “positive regulation of granulocyte macrophage colony-stimulating factor production” was less expressed in NB-pregnant heifers. The down-regulation of the gene *FCER1A* in blood samples is associated with pre-term delivery in women [55]. Another gene related to the immune system, namely *KIR3DL1*, showed the lowest transcript abundance (4-fold) in NB-pregnant compared to AI-pregnant heifers. Interestingly, recurrent miscarriage patients presented lower occurrence of *KIR3DL1* in their blood compared to healthy women [56]. When expressed in natural killer (NK) cells, KIR3DL1 inhibits [57] the cytotoxic function or the adhesive capacity of NK cells (reviewed by [58]).

We identified six DEGs in the PWBC of heifers associated with the pregnancy outcome of AI-pregnant or non-pregnant. It is critical to notice, however, that the inferences of *ALAS2*, LOC52273, *TAC3*, *TFF2* as DEGs, were mostly driven by some heifers that did not become pregnant, whereas others presented gene expression levels equivalent to the heifers that became pregnant to AI. The gene *TAC3* encodes the protein neurokinin B, whose expression is negatively regulated by ovarian derived steroids [59] and in turn stimulates the secretion of Gonadotropin-Releasing Hormone (GnRH) [60], which is has central function on the release of follicle-stimulating hormone and luteinizing hormone. On the same note, expression of the gene *CNKSR3* was upregulated by luteinizing hormone in women’s endometrium [61] and follicular granulosa cells in buffalo cows [62]. In specific heifers, the dysregulation on these two genes is suggestive of an alteration in the hormonal feedback between the ovary the hypothalamic-pituitary axis in some of the heifers that did not become pregnant.

The TSP approach compares the levels of transcript abundance for each possible pair of genes expressed within a sample [45], and it has been used as a classification or prediction tool in biomedicine [45, 63–65]. We employed this approach to evaluate the usefulness of gene expression levels in PWBC at the time of AI for classification of heifers with different pregnancy outcome. For each experimental station, the use of transcript levels for the top 20 pairs of genes clustered AI-pregnant heifers separately from the others with 100% confidence of cluster formation. Because this approach is parameter free [66, 67] with the exception of the binary variable that separates subjects into two categories, we used the algorithm to identify TSPs in all 23 samples that could identity AI-pregnant heifers. The ratio between the expression levels for four gene pairs misclassified only two out of the 12 AI-pregnant heifers.

Our investigation focused on PWBC, which are mostly composed of circulating immune cells. The immune system and female fertility are connected at many levels with the reproductive function in cattle (reviewed by Fair [34]), and circulating cells of the immune system respond to reproductive hormones [68, 69]. Our results show that specific genes have transcript abundance correlated with whether a heifer became pregnant to AI, could become pregnant later by natural breeding, or failed to become pregnant. We hypothesize that PWBC change their transcriptome as the heifers undergo the follicular phase of their estrous cycle. These changes most likely reflect the heifer’s readiness for fertilization.

The physiological relationship between the immune system of healthy heifers and their likelihood of becoming pregnant by AI is yet to be studied. In addition, further investigation is required to assess how our results may translate to other herds, especially when accounting for different management strategies, breeds, and genetic background. Although further work is needed to develop robust approaches to identify molecular markers in the transcriptome of PWBC, taken together, our results suggest a window of opportunity for the use of gene expression data as source of prognostic molecular markers of pregnancy likelihood in beef heifers.

## Conclusions

At the time of AI, specific genes expressed in PWBC displayed differential transcript abundance in heifers classified according to their pregnancy outcome (AI-, NB-, non-pregnant). This variable expression is likely associated with the heifers’ physiological condition that relates to their fertility at the time of AI. The data suggest that the heifer’s metabolic status may be critical for the AI success, and impaired hormonal regulation is among the multiple factors that may hinge the chances of pregnancy in beef heifers. Further investigation is needed to confirm these hypotheses. Using a parameter free approach, the transcript abundance of specific gene pairs distinguished most AI-pregnant, relative to NB- or non- pregnant heifers. This result showed that the transcriptome of PWBC has a promising potential to be used as a source of data to classify heifers of distinct potential to become pregnant.

## Methods

### Animal handling and heifer classification according to pregnancy outcome

Crossbred beef heifers (Angus-Simmental cross) from two Auburn University research stations (Station A: Wiregrass Research and Extension Center, *n* = 27; and Station B: Black Belt Research and Extension Center, *n* = 33) were developed to reach a target weight of 60% of their mature body weight by 13.5 months of age [13, 70]. Pre-breeding examinations were performed approximately 45 days before breeding to evaluate the pubertal status of each heifer. Reproductive tract scores (scale of 1–5; 1 = pre-pubertal, 5 = pubertal, luteal phase [16]), pelvic width, and pelvic height were determined through transrectal palpation by a single, experienced veterinarian. Additionally, heifers were evaluated for body condition score (BCS; scale of 1–9 with 1 = emaciated and 9 = obese [3]).

Heifers were then subjected to estrous synchronization for fixed-time artificial insemination with the 7-Day CO-Synch protocol. Briefly, heifers received an injection of GnRH (i.m.; 100 μg; Cystorelin®; Merial, Duluth, GA) and insertion of a CIDR (intravaginal insert; 1.38 g progesterone; Eazi-Breed® CIDR®; Zoetis Inc., Kalamazoo, MI) on day − 9, followed by CIDR removal and an injection of prostaglandin F2α (PGF; i.m.; 25 mg; Lutalyse®; Zoetis Inc., Kalamazoo, MI) on day − 2. All heifers then received a second GnRH injection (i.m.; 100 μg; Cystorelin®; Merial, Duluth, GA) and were inseminated with a dose of semen of proven fertility on day 0, 54 ± 2 h after CIDR removal and PGF injection. Two professionals were responsible for insemination procedures in both experimental stations, taking turns on random heifers.

Immediately after AI, 10 ml of blood was drawn from the jugular vein into vacutainers containing 18 mg K2 EDTA (Becton, Dickinson and Company, Franklin, NJ). The tubes were inverted for 8–10 times and immersed in ice. Upon arrival in the laboratory, the tubes were sprayed with 10% bleach and rinsed to eliminate contamination from the field. The tubes were centrifuged for 10 min at 2000×g at 4 °C. The buffy coat was removed and deposited into 14 ml of red blood cell lysis solution (0.15 M ammonium chloride, 10 mM potassium bicarbonate, 0.1 mM EDTA, Cold Spring Harbor Protocols) for 10 min at room temperature (24–25 °C). The solution was then centrifuged for 5 min at 800xg at 4C to pellet the PWBCs. The aqueous layer was discarded and the pellet was re-suspended in 200 μl of RNAlater® (Lifetechnologies™, Carlsbad, CA). The PWBCs were then stored at − 80°C prior to RNA extraction. This procedure was reproduced for both experimental stations.

Fourteen days after insemination, heifers were exposed to two fertile bulls for natural breeding for the remainder of the 86 day breeding season on station A or 42 day breeding season on station B. An experienced veterinarian performed pregnancy evaluation by transrectal palpation on day 62 and 125 post insemination at station A, and on day 95 post insemination at station B. Presence or absence of a conceptus, alongside morphological features indicating fetal age were recorded, and heifers were classified as pregnant to AI, pregnant to natural service, or non-pregnant. Heifers that became pregnant after the first 21 days of breeding were identified as late breeding for the purpose of this study.

### Selection of heifers for RNA-sequencing of PWBC

Eleven heifers (six AI-pregnant and five NB-pregnant) were selected from station A, and twelve heifers (six AI-pregnant and six non-pregnant) were selected from station B for RNA-sequencing. Within station, heifers were selected according to their similarities of age and phenotypic parameters. Data for age, weaning weight, pelvic height, pelvic width, and pelvic area were compared between groups using Krustal-Wallis rank sum test. Body condition and reproductive tract scores were tested using Fisher’s exact test. Tests were conducted in R software. Selected heifers did not differ for phenotypic traits associated with puberty (Additional file 1: Table S1), and all heifers were of pubertal status at the time of breeding. The selection of heifers from different groups that were phenotypically similar, according to trait average and standard deviation, avoided the addition of covariates in the analysis of differential gene expression.

### RNA extraction, library preparation, and RNA sequencing

Total RNA was then isolated from PWBCs of 23 heifers using TRIzol™ reagent (Invitrogen, Carlsbad, CA) following the manufacturer’s protocol. RNA yield was quantified using the Qubit™ RNA Broad Range Assay Kit (Eurogene, OR) on a Qubit® Fluorometer, and integrity was assessed on Agilent 2100 Bioanalyzer (Agilent, Santa Clara, CA) using an Agilent RNA 6000 Nano kit (Agilent, Santa Clara, CA). We obtained RIN values ranging between 7.7 and 8.8. Furthermore, samples with rRNA ratios (28S:18S) greater than 1.5 were further processed for library construction (range 1.5–1.8). Libraries were prepared with the TruSeq Stranded mRNA Library Prep kit (Illumina, Inc., San Diego, CA) following manufacturer’s instructions. Libraries were quantified with Qubit™ dsDNA High Sensitivity Assay Kit (Eurogene, OR) and quality was evaluated using the High Sensitivity DNA chip (Agilent, Santa Clara, CA) on an Agilent 2100 Bioanalyzer (Agilent, Santa Clara, CA). Libraries were sequenced in a HiSeq 2500 system at the Genomic Services Laboratory at HudsonAlpha, Huntsvile, AL to generate 125 nucleotide long pair-end reads.

### RNA sequencing data processing

Sequences were trimmed of their adapters and submitted to a custom build bioinformatics pipeline [71]. Reads were aligned to the bovine genome (UMD3.1 [37]), and sequences aligning to multiple places on the genome, with 5 or more mismatches were filtered out. The sequences were then marked for duplicates, and non-duplicated pairs of reads were used for gene expression study. The read-pairs were counted against the Ensembl gene annotation [39] (version 1.87) using HTSeq [72].

### Differentially expressed genes

Differences of transcript levels between samples at each experimental station were determined from fragment counts [38] using the Bioconductor packages “edgeR” [73] and “DESeq2” [44] in R software [74]. Genes were considered detected if the counts per million was greater than one in six or more samples. For each experimental station, a gene was inferred as differentially expressed if the nominal *P* value was ≤ 0.01. This nominal P value corresponded to empirical false discovery rate (eFDR) of 0.02 for station A and 0.05 for station B (Additional file 1: Figure S1), as calculated according to the procedure outlined elsewhere [75], using 10,000 randomizations of sample classification.

### Validation of DEGs

We used RNA extracted from the PWBC of the 23 heifers from station A and B whose PWBC transcriptome was evaluated though RNA sequencing to confirm the DEGs by RT-qPCR. We synthesized complementary DNA from 500 ng of total RNA and using oligodT_15_ (Promega, Madison, WI). Reverse transcription was carried out with SuperScriptII (Invitrogen, Carlsbad, CA) following manufacturer’s recommendations. The final RT reaction was diluted 1:2 (v:v) and 1μl was used as template for each PCR reaction using Perfecta SYBR Green FastMix (Quanta Biosciences), and 100 nM of each primer (Additional file 1: Table S3, IDT) in a final volume of 10μl. Primers were designed using PrimerBlast application following the recommendations for obtaining target-specific primers for PCR [76]. The reactions were assayed in a Roche Light Cycler 480 equipment (Roche) equipment with pre-incubation at 95 °C for 1 min, followed by 40 cycles of 95 °C for 15 s and 60 °C for 45 s. A melting curve was generated using the thermocycler’s default parameters to validate the presence of a unique amplicon. The identification of unique amplicon is a proxy of primer specificity. Primer efficiency and cycle threshold (CT) was determined for all reactions using the LinRegPCR program [77].

We used *GAPDH* as a reference gene, which presented similar Ct values across all samples (Additional file 1: Figure S4) and showed no difference of transcript abundance between the groups tested (*P* > 0.9, t-test, Additional file 1: Table S4). The ΔCT was calculated for each corresponding target gene relative to the reference gene, and the values of ΔCT were used as input for a t-test to assess the significance of differences between the two groups [78]. We inferred that the averages of gene expression levels were statistically different when *P* ≤ 0.1. We adopted alpha = 0.1 for qPCR analysis because comparing normalized gene expression levels between groups with six samples in each group presents the power of 0.65 to detect an effect of 1 at the significance level of 0.1.

### Pairs of genes with expression ratios indicating fertility categorization

Fragments per kilobase per million reads (FPKM) were calculated using the function “rpkm()” from “edgeR”. FPKM was the used as input for the calculation of TSP using the package “tspair” [66]. The TSP approach [45] identifies genes whose transcript abundance ratios within each individual can classify subjects into binary categories. The ratios of the 20 TSP were used as input for hierarchical clustering of the samples, and the robustness of the clusters was calculated using 5000 randomizations with the R package “pvclust” [79].

## Additional files

Additional file 1: Supplementary figures and tables. (DOCX 639 kb)

Additional file 2: R Code that reproduces the RNA-seq analyses performed in this study. (PDF 1676 kb)

## Abbreviations

AI: Artificial insemination
BCS: Body condition score
CIDR: Controlled internal drug release
CPM: Count per million
CT: Cycle threshold
DEG: Differentially expressed gene
FDR: False discovery rate
FPKM: Fragment per kilobase per million reads
GnRH: Gonadotropin releasing hormone
N: Number
P: Probability
PGF: Prostaglandin F2α
PM: Pelvic measurements
PWBC: Peripheral white blood cells
qPCR: Quantitative real-time polymerase chain reaction
RNA: Ribonucleic acid
RT: Reverse transcription
RTS: Reproductive tract score
TSP: Top scoring pairs
