# Supplementary material for: Transcriptome profiles in peripheral white blood cells at the time of artificial insemination discriminate beef heifers with different fertility potential
Source: BMC Genomics. 2018 Feb 9;19:129. doi: 10.1186/s12864-018-4505-4 (PMC5807776; doi:10.1186/s12864-018-4505-4)
Supplement: Supplementary file 1 — Supplementary figures and tables. (DOCX 639 kb) [file 12864_2018_4505_MOESM1_ESM.docx]

b

a

⏺edgeR ⏶DESeq2

Figure S1. Correspondence between nominal P values and empirical FDR. (a) Comparison of gene expression levels between AI-pregnant, NB-pregnant in experimental station A. (b) Comparison of gene expression levels between AI-pregnant, non-pregnant in the experimental station B.

a

continue on next page…

b

Figure S2. Scatterplots of expression levels (FPKM) of the top scoring pairs that distinguish heifers according to pregnancy outcome. Top 20 TSPs for Station A (a) and B (b).

Figure S3. Scatterplots of expression levels (FPKM) of 20 randomly chosen pairs of genes expressed in heifers from station A that did not form top scoring pairs. The charts represent the null hypothesis for the TSP approach

Table S1. Descriptive statistics ($\bar{x}\pm\hat{\sigma})$ of the phenotypical data from the heifers used for the sequencing analysis.

| Location | Parameter | Pregnant AI | Pregnant NB | P |
| --- | --- | --- | --- | --- |
| A | N | 6 | 5 | - |
|  | Age^a^ | 412 $\pm$ 10 | 402 $\pm$ 11 | 0.36^d^ |
|  | WW^b^ | 234.6 $\pm$ 14.3 | 249.5 $\pm$ 21.2 | 0.14^d^ |
|  | Pelvic height^c^ | 15.3 $\pm$ 0.5 | 15.0 $\pm$ 0.7 | 0.39^d^ |
|  | Pelvic width^c^ | 11.3 $\pm$ 0.5 | 11.6 $\pm$ 1.5 | 1 |
|  | Pelvic area^c^ | 173.8 $\pm$ 11.0 | 174.8 $\pm$ 31 | 0.8^d^ |
|  | BCS | 5.7 $\pm$ 0.5 | 5.8 $\pm$ 0.4 | 1^e^ |
|  | RTS | 4.7 $\pm$0.5 | 4.2 $\pm$ 0.8 | 0.7^e^ |
| Location | Parameter | Pregnant AI | Not Pregnant | P |
| B | N | 6 | 6 | - |
|  | Age^a^ | 428 $\pm$ 8 | 433 $\pm$ 10 | 0.4^d^ |
|  | WW^b^ | 325.8 $\pm$ 15.4 | 316.4 $\pm$ 15.4 | 0.5^d^ |
|  | BCS | 6 $\pm$ 0 | 6 $\pm$ 0 | - |
|  | RTS | 4.5 $\pm$ 0.5 | 4.2 $\pm$ 0.4 | 0.5^e^ |

BCS: body condition score; RTS: reproductive tract score; WW: weaning weight; ^a^ day; ^b^ kg; ^c^ cm^2^; ^d^ Krustal-Wallis rank sum test; ^e^ Fisher’s exact test; - Statistical test is not applicable.

Table S2. Number of read-pairs generated and aligned uniquely to the *Bos taurus* reference genome UMD 3.1.

| Sample | N pairs sequenced | N pairs aligned | Alignment (%) |
| --- | --- | --- | --- |
| SL220764 | 36,128,269 | 30,457,166 | 84.3 |
| SL220765 | 36,260,080 | 29,334,579 | 80.9 |
| SL220766 | 19,966,449 | 17,252,337 | 86.4 |
| SL220767 | 22,775,841 | 18,158,658 | 79.7 |
| SL220768 | 14,805,290 | 12,403,405 | 83.8 |
| SL220769 | 45,845,646 | 39,345,350 | 85.8 |
| SL220771 | 16,970,202 | 13,927,564 | 82.1 |
| SL220772 | 18,827,465 | 14,700,579 | 78.1 |
| SL220773 | 18,309,120 | 15,031,613 | 82.1 |
| SL220774 | 21,112,166 | 17,454,399 | 82.7 |
| SL220775 | 32,485,522 | 27,287,566 | 84.0 |
| SL253803 | 27,341,041 | 24,446,159 | 89.4 |
| SL253804 | 25,477,964 | 22,971,157 | 90.2 |
| SL253805 | 15,799,927 | 14,684,897 | 92.9 |
| SL253806 | 18,093,354 | 16,667,914 | 92.1 |
| SL253807 | 24,358,249 | 22,330,930 | 91.7 |
| SL253808 | 18,861,053 | 16,636,677 | 88.2 |
| SL253809 | 18,352,301 | 16,562,097 | 90.2 |
| SL253810 | 21,052,090 | 18,776,453 | 89.2 |
| SL253811 | 41,238,769 | 36,636,894 | 88.8 |
| SL253812 | 18,757,099 | 16,756,241 | 89.3 |
| SL253813 | 24,846,720 | 22,760,158 | 91.6 |
| SL253814 | 19,575,486 | 16,879,869 | 86.2 |
| Average | 24,227,831 | 20,933,159 | 86.5 |

Table S3. Validation of RNA-sequencing results contrasting the gene expression in PWBC between heifers with different pregnancy outcome.

| Symbol | RNA-seq |  | qPCR | | mRNA | Oligonucleotides | Amplicon | x̅ PCR |
| --- | --- | --- | --- | --- | --- | --- | --- | --- |
|  | FC^a^ |  | FC^a^ | P | Accession # |  | (bp^b^) | Efficiency |
| *GAPDH* | * |  | - | - | NM_001034034.2 | TGGTGAAGGTCGGAGTGAAC | 91 | 1.9 |
|  |  |  |  |  |  | ATGGCGACGATGTCCACTTT |  |  |
| **Location A** | | | | | | | | |
| *ALDH5A1* | 1.23 |  | 2.50 | 0.04 | NM_001192735.1 | CCCCAGCAAAAGAAAGGCG | 99 | 1.9 |
|  |  |  |  |  |  | CTTCCGTGTGATCATGGCACT |  |  |
| *FCER1A* | -1.15 |  | -1.74 | 0.02 | NM_001100310.2 | GTGGGCAGAATTCAGAGGCT | 88 | 1.9 |
|  |  |  |  |  |  | GCCAGAAATAGTTGCTTTGAGGG |  |  |
| *TTLL1* | 1.19 |  | 2.00 | 0.02 | NM_001076171.1 | AGAAGGACGAAAGCGGGAAG | 79 | 1.9 |
|  |  |  |  |  |  | AACAGGTTGTAGTCGGCAGG |  |  |
| *SIGLEC14* | -1.22 |  | -1.75 | 0.09 | XM_015458276.1 | TCCGGCTCAACGTCTCCTAT | 98 | 1.9 |
|  |  |  |  |  |  | CTCCAGGATGGGCAGTGAC |  |  |
| **Location B** | | | | | | | | |
| *TAC3* | 2.17 |  | 3.04 | 0.05 | NM_181017.2 | GCACCTTCAAGTACCCTCCA | 70 | 1.9 |
|  |  |  |  |  |  | TCTTCACGATGTAGCCCAGG |  |  |
| LOC522763 | 1.92 |  | 8.77 | 0.10 | NM_001102069.1 | GCACCGAGCTCTTGACTGAT | 118 | 1.9 |
|  |  |  |  |  |  | GTGAAGGCTGAAGCTCAGGA |  |  |

^a^ FC: Fold change (pregnant NB/pregnant AI(Location A); non-pregnant/pregnant AI (Location B)), ^b^ bp: base pairs.

* *GAPDH* fold change in Station A: 0.7 and Station B: 0.9

Station A Station B

1.9^-Ct^

Preg AI Preg NB Preg AI not Preg

Figure S4. Real time polymerase chain reaction data points for *GAPDH* transcripts. Please, see discussion in Livak and Schmittgen [1] and in Schmittgen and Livak [2] for the rationale on plotting the data as 1.9^-Ct^.

Table S4. Averages of real time polymerase chain reaction data points (2^-Ct^) for *GAPDH* transcripts and summary of statistical tests to compare the averages.

| Station A | Preg AI | Preg NT | F | P_(F test)_ | t | P_(t test)_ |
| --- | --- | --- | --- | --- | --- | --- |
|  | 1.5E-06 | 1.6E-06 | 0.5950 | 0.1471 | -0.0981 | 0.9224 |
| Station B | Preg AI | not Preg | F | P_(F test)_ | t | P_(t test)_ |
|  | 3.9E-06 | 3.9E-06 | 0.8207 | 0.3492 | 0.0412 | 0.9674 |

1. Livak KJ, Schmittgen TD: **Analysis of relative gene expression data using real-time quantitative PCR and the 2(T)(-Delta Delta C) method**. *Methods* 2001, **25**(4):402-408.

2. Schmittgen TD, Livak KJ: **Analyzing real-time PCR data by the comparative C(T) method**. *Nat Protoc* 2008, **3**(6):1101-1108.
