# Supplementary material for: Transcriptome profiles in peripheral white blood cells at the time of artificial insemination discriminate beef heifers with different fertility potential
Source: BMC Genomics. 2018 Feb 9;19:129. doi: 10.1186/s12864-018-4505-4 (PMC5807776; doi:10.1186/s12864-018-4505-4)
Supplement: Supplementary file 2 — R Code that reproduces the RNA-seq analyses performed in this study. (PDF 1676 kb) [file 12864_2018_4505_MOESM2_ESM.pdf]

Transcriptome profiles in peripheral white blood cells at the time of artificial insemination discriminate beef heifers with different fertility potential

Sarah E Dickinson, Brock Griffin, Michelle Elmore, Lisa Kriese-Anderson, Joshua Elmore, Paul Dyce, Soren Rodning, Fernando H Biase

December/2017

Contents

Overview 2

Load the libraries 2

DEGs for STATION A 3

Load count data 3

DEGs for STATION B 8

Load count data 8

Pannel Figure 2 14
- Fig 2a . . . 14
- Fig 2b,c . . . 14
- Fig 2d,e . . . 16

TSP for STATION A 19
- Load the filtered FPKM data . . . 19

TSP for STATION B 20
- Load the filtered FPKM data . . . 20

TSP for STATIONS A & B 20

Pannel Figure 3 20
- Fig 3a . . . 20
- Fig 3b . . . 23
- Fig 3c . . . 25
- Fig 3d . . . 27
- Fig 3e . . . 30

Code created by Fernando Biase and Sarah Dickinson.

Please, direct questions to Fernando Biase at *fbiase* at *auburn* dot *edu*.

Overview

The code presented below allows the readers to reproduce the results we obtained in our paper Dickinson et al. 2017. Transcriptome profiles in peripheral white blood cells at the time of artificial insemination discriminate beef heifers with different fertility potential.

Please, see paper for details on heifer classification and the contrasts performed for the inference of differentially expressed genes.

The count and FPKM data to reproduce this code can be found on the NCBI GEO public repository GSE103628

Alternatively, the files needed to reproduce the codes below can be downloaded using the follwoing links:

2017_08_25_count_station_A.txt.bz2

2017_09_07_count_station_B.txt.bz2

2017_09_07_FPKM_filtered_station_A.txt.bz2

2017_09_07_FPKM_filtered_station_B.txt.bz2

2017_08_19_empirical_FDR_stationA.txt

2017_08_19_empirical_FDR_stationB.txt

multiplot.R

Load the libraries

```
library("ggplot2")
library("reshape2")
library("org.Bt.eg.db")
library("VennDiagram")
library("annotate")
library("doParallel")
library("gtools")
library("bigmemory")
library("biomaRt")
library("edgeR")
library("DESeq2")
library("gridExtra")
library("gplots")
library("Heatplus")
library("pvclust")
library("RColorBrewer")
library("ComplexHeatmap")
library("tspair")
library("stats")
library("circlize")
library("knitr")
library("dendextend")
source("multiplot.R") #from Cookbook for R Multiple graphs on one page
#(http://www.cookbook-r.com/Graphs/Multiple_graphs_on_one_page_%28ggplot2%29/)
```

DEGs for STATION A

Load count data

```
count_unfiltered_stationA<-
  read.delim('2017_08_25_count_station_A.txt.bz2',
             row.names=1,header =TRUE,stringsAsFactors =FALSE)
```

	SL220764	SL220765	SL220766	SL220767	SL220768	SL220769	SL220771	SL220772
ENSBTAG00000000005	1674	381	876	96	748	2357	98	142
ENSBTAG00000000008	18	4	8	2	8	32	2	6
ENSBTAG00000000009	1	1	0	0	0	0	0	1
ENSBTAG00000000010	1191	1078	708	653	453	1444	396	696
ENSBTAG00000000011	0	3	1	1	0	5	0	1
ENSBTAG00000000012	262	148	163	43	114	357	100	73

Analyze the data using the edgeR package

```
group<-factor(c("preg_AI","preg_AI","preg_AI","preg_bull","preg_bull","preg_bull",
                "preg_AI","preg_AI","preg_bull","preg_bull","preg_AI"),
              levels=c("preg_AI","preg_bull"))

design <- model.matrix(~group)

design <- model.matrix(~group)
dds<-DGEList(count=count_unfiltered_stationA, group=group)
keep<-rowSums(cpm(dds)>1) >=6
dds<-dds[keep, keep.lib.sizes=FALSE]
dds<-estimateDisp(dds, design, robust=TRUE)
dds <- glmFit(dds, design)
dds <- glmLRT(dds)
stationA_res_preg_bull_preg_AI_edgeR<-topTags(dds,adjust.method="none",n=Inf)$table
```

	logFC	logCPM	LR	PValue
ENSBTAG00000047764	-4.1466862	3.8954847	20.004583	0.0000077
ENSBTAG00000021902	1.2020919	4.9492775	14.375406	0.0001497
ENSBTAG00000001842	1.7636469	0.3144043	13.435639	0.0002469
ENSBTAG00000022715	1.9810638	1.6186805	13.343041	0.0002594
ENSBTAG00000035868	-1.3084380	2.9102587	13.260297	0.0002711
ENSBTAG00000000271	1.2831237	1.6220746	12.886935	0.0003309
ENSBTAG00000026779	-0.6658679	9.9687268	10.323672	0.0013133
ENSBTAG00000012030	1.3598201	6.6160028	9.376862	0.0021974
ENSBTAG00000045492	-1.1112499	3.8251913	9.089231	0.0025712
ENSBTAG00000040367	-1.4141116	5.4186008	8.692626	0.0031950

Analyze the data using the DESeq2 package

```
group<-factor(c("preg_AI","preg_AI","preg_AI","preg_bull","preg_bull","preg_bull",
                "preg_AI","preg_AI","preg_bull","preg_bull","preg_AI"),
              levels=c("preg_AI","preg_bull"))

design <- model.matrix(~group)
colData<-data.frame("group"=group)
rownames(colData)<-colnames(count_unfiltered_stationA)
count_filtered_stationA<-count_unfiltered_stationA[keep,]
dds<-DESeqDataSetFromMatrix(countData=count_filtered_stationA, colData=colData,
                            design= ~group)
dds <- DESeq(dds)
stationA_res_preg_bull_preg_AI_DESeq <- results(dds,
                                               contrast=c("group","preg_bull","preg_AI"),
                                               pAdjustMethod="none", tidy=TRUE)
stationA_res_preg_bull_preg_AI_DESeq<-
  stationA_res_preg_bull_preg_AI_DESeq[with(stationA_res_preg_bull_preg_AI_DESeq,
                                            order(pvalue)), ]
```

	row	baseMean	log2FoldChange	lfcSE	stat	pvalue	padj
12019	ENSBTAG00000047764	108.000614	-3.7915866	0.7855666	-4.826563	0.0000014	0.0000014
10826	ENSBTAG00000035868	58.355987	-1.1872286	0.2532883	-4.687262	0.0000028	0.0000028
9604	ENSBTAG00000021902	272.954181	1.4389524	0.3421889	4.205140	0.0000261	0.0000261
124	ENSBTAG00000000271	25.850820	1.5155057	0.3874509	3.911478	0.0000917	0.0000917
9717	ENSBTAG00000022715	25.896057	2.1461237	0.5607438	3.827281	0.0001296	0.0001296
11547	ENSBTAG00000045492	111.594449	-0.9485599	0.2709739	-3.500558	0.0004643	0.0004643
5694	ENSBTAG00000012887	102.761332	-1.1751591	0.3452697	-3.403597	0.0006650	0.0006650
8295	ENSBTAG00000018942	307.460116	0.8631319	0.2560977	3.370323	0.0007508	0.0007508
9266	ENSBTAG00000021077	1224.551778	-3.4572436	1.0445538	-3.309780	0.0009337	0.0009337
832	ENSBTAG00000001842	9.673775	1.8706529	0.5818850	3.214815	0.0013053	0.0013053

Merge the results obtained from edger.

```
stationA_merged_res_preg_bull_preg_AI<-
  merge(stationA_res_preg_bull_preg_AI_edgeR, stationA_res_preg_bull_preg_AI_DESeq,
        by.x="row.names", by.y="row")
```

Row.names	logFC	logCPM	LR	PValue	baseMean	log2FoldChange	lfcSE	[illegible]
ENSBTAG00000000005	-0.0020491	5.889410	0.0000108	0.9973744	445.1964	0.0720871	0.5100643	[illegible]
ENSBTAG00000000010	-0.0615149	6.425524	0.0686832	0.7932638	744.9715	0.1413221	0.3153779	[illegible]
ENSBTAG00000000012	-0.1743195	3.952466	0.3146647	0.5748318	127.0515	-0.0512721	0.2377229	[illegible]
ENSBTAG00000000013	0.0364942	6.310271	0.0230140	0.8794207	656.2608	0.1877259	0.1364270	[illegible]
ENSBTAG00000000014	-0.1233633	6.409366	0.3721777	0.5418196	712.1276	0.0591350	0.1620754	[illegible]

We infered that a gene was differentially expressed if the nominal P value was lower or equal than 0.01 on the result obtained from both packages.

```
stationA_merged_res_preg_bull_preg_AI_sig<-
  stationA_merged_res_preg_bull_preg_AI[(stationA_merged_res_preg_bull_preg_AI$PValue <=0.01 &
                                         stationA_merged_res_preg_bull_preg_AI$pvalue <=0.01),]
```

	Row.names	logFC	logCPM	LR	PValue	baseMean	log2FoldChange	lfcS
124	ENSBTAG00000000271	1.283124	1.6220746	12.886935	0.0003309	25.850820	1.515506	0.387450
832	ENSBTAG00000001842	1.763647	0.3144043	13.435639	0.0002469	9.673775	1.870653	0.581885
2042	ENSBTAG00000004574	1.127263	0.8958067	7.152469	0.0074861	14.661323	1.354615	0.452773
2677	ENSBTAG00000006035	-1.845290	6.5981262	7.967110	0.0047635	784.096472	-1.843198	0.594933
5326	ENSBTAG00000012030	1.359820	6.6160028	9.376862	0.0021974	897.697143	1.621392	0.510377

Annotate the DEGs

```
mart <- useMart("ensembl")
datasets <- listDatasets(mart)
mart_bov<-useDataset("btaurus_gene_ensembl",mart)
```

```
annotation_ensembl_biomart<-getBM(attributes = c("ensembl_gene_id","hgnc_symbol",
                                                 "external_gene_name",
                                                 "gene_biotype", "description"),
                                  filters = "ensembl_gene_id",
                                  values = unique(stationA_merged_res_preg_bull_preg_AI_sig$Row.names),
                                  mart = mart_bov)

stationA_merged_res_preg_bull_preg_AI_sig_annotated<-
  merge(stationA_merged_res_preg_bull_preg_AI_sig,annotation_ensembl_biomart,
        by.x="Row.names", by.y="ensembl_gene_id")
```

Row.names	logFC	logCPM	LR	PValue	baseMean	log2FoldChange	lfcSE	
ENSBTAG00000000271	1.283124	1.6220746	12.886935	0.0003309	25.850820	1.515506	0.3874509	3.9
ENSBTAG00000001842	1.763647	0.3144043	13.435639	0.0002469	9.673775	1.870653	0.5818850	3.2
ENSBTAG00000004574	1.127263	0.8958067	7.152469	0.0074861	14.661323	1.354615	0.4527732	2.9
ENSBTAG00000006035	-1.845290	6.5981262	7.967110	0.0047635	784.096472	-1.843198	0.5949331	-3.0
ENSBTAG00000012030	1.359820	6.6160028	9.376862	0.0021974	897.697143	1.621392	0.5103779	3.1

Calculating the eFDR

The calculation of the empirical FDR (eFDR) follows the principles and formula presented by Storey and Tibshirani (2003) PNAS, with a modification described in Sham and Purcell (2014) Nature Reviews Genetics.

The following code chunk was prepared for a multicore computer, and was not run here. Instead we upload the files and build figures produced from the files used for the paper.

```
permutation_matrix<-matrix(,nrow=200000,ncol=11)

for (i in seq(1:200000)){
  sampling <- sample(1:11,11, replace = FALSE)
  if ( ! identical(sampling , c(1:11))){
    permutation_matrix[i,]<-sample(1:11,11, replace = FALSE)
  }}

permutation_matrix<-permutation_matrix[!duplicated(permutation_matrix),]
dim(permutation_matrix)
permutation_matrix<-permutation_matrix[sample(1:199496 , 10000),]
rand<-dim(permutation_matrix)[1]
```

```r
sequence.pvalue<-seq(0.005, 0.05, 0.005)

results <- filebacked.big.matrix(length(sequence.pvalue),rand, type="double",
                                    init=0, separated=FALSE,
      backingfile="incidence_matrix.bin",
      descriptor="incidence_matrix.desc")
mdesc_result<- describe(results)

#calculate eFDR for edger

cl <- makeCluster(10)
registerDoParallel(cl)

results[,]<-foreach(i = sequence.pvalue, .combine='rbind', .inorder=TRUE,
                    .packages=c("edgeR","bigmemory"), .verbose=TRUE) %:%
  foreach(j = 1:rand, .combine='cbind', .inorder=FALSE,.packages=c("edgeR","bigmemory"),
          .verbose=TRUE ) %dopar% {
    group<-c("preg_AI","preg_AI","preg_AI","preg_bull","preg_bull",
             "preg_bull","preg_AI","preg_AI","preg_bull","preg_bull",
             "preg_AI")
    group<-group[permutation_matrix[j,]]
    group<-factor(group, levels=c("preg_AI","preg_bull"))
    design <- model.matrix(~group)
    dds<-DGEList(count=count_unfiltered_stationA, group=group)
    keep<-rowSums(cpm(dds)>1) >=6
    dds<-dds[keep, , keep.lib.sizes=FALSE]
    dds<-estimateDisp(dds, design, robust=TRUE)
    dds <- glmFit(dds, design)
    dds <- glmLRT(dds)
    #topTags(dds,n=Inf)$table$PValue

    length(which(topTags(dds,n=Inf)$table$PValue <= i))
          }

stopCluster(cl)

total.rand <- rand * dim(count_pwbc_pairend1)[1]

qvalue<-data.frame(raw.pvalue = sequence.pvalue,
                     e.pvalue.edgeR= (rowSums(results[,]+1))/(total.rand+1),
                   e.pvalue.edgeR.round= round((rowSums(results[,]+1))/(total.rand+1)
                                               ,4))


rm(results)
system("rm incidence_matrix.bin")
system("rm incidence_matrix.desc")


#calculate eFDR for DESeq2

results.rand <- filebacked.big.matrix(length(sequence.pvalue),rand, type="double",
                                      init=0, separated=FALSE,
```

```
                                backingfile="incidence_matrix.bin",
                                descriptor="incidence_matrix.desc")
mdesc_result<- describe(results.rand)

cl <- makeCluster(10)
registerDoParallel(cl)

results.rand[,]<-foreach(i = sequence.pvalue, .combine='rbind', .inorder=TRUE,
                         .packages=c("DESeq2","bigmemory"), .verbose=TRUE) %:%

  foreach(j = 1:rand, .combine='cbind', .inorder=FALSE,.packages=c("DESeq2","bigmemory"),
          .verbose=TRUE ) %dopar% {

   group<-c("preg_AI","preg_AI","preg_AI","preg_bull","preg_bull",
             "preg_bull","preg_AI","preg_AI","preg_bull","preg_bull",
             "preg_AI")
   group<-group[permutation_matrix[j,]]
   group<-factor(group, levels=c("preg_AI","preg_bull"))
   design <- model.matrix(~group)
   rownames(colData)<-colnames(count_unfiltered_stationA)
   count_filtered_stationA<-count_unfiltered_stationA[keep,]
   dds<-DESeqDataSetFromMatrix(countData=count_filtered_stationA, colData=colData,
                               design= ~group)
   dds <- DESeq(dds)
   stationA_res_preg_bull_preg_AI_DESeq <- results(dds,
                                                   contrast=c("group","preg_bull" ,"preg_AI"),
                                                   pAdjustMethod="none", tidy=TRUE)
  length(which(stationA_res_preg_bull_preg_AI_DESeq$pvalue <= i))
  }

stopCluster(cl)

total.rand <- rand * dim(count_pwbc_pairend1)[1]

qvalue2<-data.frame(raw.pvalue = sequence.pvalue,
                   e.pvalue.DESEQ2= (rowSums(results.rand[,]+1))/(total.rand+1),
                   e.pvalue.DESEQ2.round= round((rowSums(results.rand[,]+1))/(total.rand+1),
                                                4))

qvalue3<-cbind(qvalue,qvalue2)

rm(results.rand)
system("rm incidence_matrix.bin")
system("rm incidence_matrix.desc")

write.table(qvalue3, file="2017_08_19_empirical_FDR_stationA.txt",
            quote=FALSE, sep="\t", row.names=FALSE)
```

DEGs for STATION B

Load count data

```
count_unfiltered_stationB<-
  read.delim('2017_09_07_count_station_B.txt.bz2',row.names=1,header =TRUE,
             stringsAsFactors =FALSE)
```

	SL253803	SL253804	SL253805	SL253806	SL253807	SL253808	SL253809	SL253810
ENSBTAG00000000005	861	1188	560	395	996	818	513	770
ENSBTAG00000000008	12	9	7	7	8	5	4	5
ENSBTAG00000000009	0	0	0	0	0	1	1	0
ENSBTAG00000000010	149	893	82	109	155	645	118	209
ENSBTAG00000000011	1	0	1	0	2	1	0	2
ENSBTAG00000000012	354	262	214	224	412	160	282	341

Analyze the data using the edgeR package

```
rm(group,design,keep)
group<-factor(c("preg_AI","not_preg","not_preg","not_preg","preg_AI","preg_AI",
                "preg_AI","preg_AI","not_preg","preg_AI","not_preg","not_preg"),
              levels=c("preg_AI","not_preg"))
design <- model.matrix(~group)
dds<-DGEList(count=count_unfiltered_stationB, group=group)
keep<-rowSums(cpm(dds)>1) >=6
dds<-dds[keep, keep.lib.sizes=FALSE]
dds<-estimateDisp(dds, design, robust=TRUE)
dds <- glmFit(dds, design)
dds <- glmLRT(dds)
stationB_res_not_preg_preg_AI_edgeR<-topTags(dds,adjust.method="none",n=Inf)$table
```

	logFC	logCPM	LR	PValue
ENSBTAG00000030814	3.4199039	3.3357534	10.828640	0.0009994
ENSBTAG00000021807	2.1697006	5.6998375	8.862451	0.0029110
ENSBTAG00000003414	-0.9760004	0.8983029	7.142139	0.0075293
ENSBTAG00000012674	1.2764692	1.0871751	7.118853	0.0076277
ENSBTAG00000001308	1.9151902	3.6978275	6.895329	0.0086421
ENSBTAG00000013178	1.8233097	3.2171654	6.831173	0.0089580
ENSBTAG00000021151	0.8551591	1.6224304	6.632162	0.0100154
ENSBTAG00000027962	1.6706396	2.5016212	6.453460	0.0110737
ENSBTAG00000013869	1.1292859	1.5287651	6.190799	0.0128416
ENSBTAG00000046273	1.0466245	1.2931427	6.155359	0.0131015

Analyze the data using the DESeq2 package

```
group<-factor(c("preg_AI","not_preg","not_preg","not_preg","preg_AI","preg_AI",
                "preg_AI","preg_AI","not_preg","preg_AI","not_preg","not_preg"),
```

```
              levels=c("preg_AI","not_preg"))
design <- model.matrix(~group)
colData<-data.frame("group"=group)
rownames(colData)<-colnames(count_unfiltered_stationB)
count_filtered_stationB<-count_unfiltered_stationB[keep,]
dds<-DESeqDataSetFromMatrix(countData=count_filtered_stationB,
                            colData=colData, design= ~group)
dds <- DESeq(dds)
stationB_res_not_preg_preg_AI_DESeq <- results(dds, contrast=c("group","not_preg" ,"preg_AI"),
                                               pAdjustMethod="none", tidy=TRUE)
stationB_res_not_preg_preg_AI_DESeq<-
  stationB_res_not_preg_preg_AI_DESeq[with(stationB_res_not_preg_preg_AI_DESeq, order(pvalue)), ]
```

	row	baseMean	log2FoldChange	lfcSE	stat	pvalue	padj
9219	ENSBTAG00000030814	88.13271	3.4731204	0.9598317	3.618468	0.0002964	0.0002964
6520	ENSBTAG00000016522	416.04060	-0.4300282	0.1313069	-3.274987	0.0010567	0.0010567
1387	ENSBTAG00000003414	14.43022	-1.0093576	0.3187810	-3.166304	0.0015439	0.0015439
8332	ENSBTAG00000021151	24.82570	0.8467125	0.2716848	3.116525	0.0018300	0.0018300
8566	ENSBTAG00000021807	455.80019	2.1960477	0.7096633	3.094492	0.0019715	0.0019715
7555	ENSBTAG00000019225	251.06310	0.3407394	0.1166038	2.922198	0.0034757	0.0034757
6022	ENSBTAG00000015193	171.88666	-0.8705358	0.3043331	-2.860471	0.0042301	0.0042301
523	ENSBTAG00000001308	111.89445	1.8992808	0.6933315	2.739355	0.0061560	0.0061560
2050	ENSBTAG00000005077	85.74071	0.6217035	0.2275713	2.731906	0.0062969	0.0062969
5010	ENSBTAG00000012674	16.58941	1.2570446	0.4605126	2.729664	0.0063399	0.0063399

Merge the results obtained from edger.

```
stationB_merged_res_not_preg_preg_AI<-
  merge(stationB_res_not_preg_preg_AI_edgeR, stationB_res_not_preg_preg_AI_DESeq,
        by.x="row.names", by.y="row")
```

Row.names	logFC	logCPM	LR	PValue	baseMean	log2FoldChange	lfcSE	
ENSBTAG00000000005	-0.0700480	6.408825	0.0681456	0.7940562	739.7220	-0.0850549	0.2035330	[illegible]
ENSBTAG00000000010	0.5512007	5.145658	0.7712566	0.3798286	309.5856	0.5641332	0.6242998	[illegible]
ENSBTAG00000000012	-0.2461390	5.104157	0.4062598	0.5238740	297.5895	-0.2783346	0.3047471	[illegible]
ENSBTAG00000000013	-0.2301179	7.734369	0.3331460	0.5638124	1854.9386	-0.2679968	0.4068876	[illegible]
ENSBTAG00000000014	0.7037463	5.103830	0.8929252	0.3446858	300.4954	0.7242494	0.7450302	[illegible]

We infered that a gene was differentially expressed if the nominal P value was lower or equal than 0.01 in the result obtained from both packages.

```
stationB_merged_res_not_preg_preg_AI_sig<-
  stationB_merged_res_not_preg_preg_AI[(stationB_merged_res_not_preg_preg_AI$PValue <=0.01 &
                                         stationB_merged_res_not_preg_preg_AI$pvalue <=0.01),]
```

	Row.names	logFC	logCPM	LR	PValue	baseMean	log2FoldChange	lfcSE
523	ENSBTAG00000001308	1.9151902	3.6978275	6.895329	0.0086421	111.89445	1.899281	0.6933315
1387	ENSBTAG00000003414	-0.9760004	0.8983029	7.142139	0.0075293	14.43022	-1.009358	0.3187810
5010	ENSBTAG00000012674	1.2764692	1.0871751	7.118853	0.0076277	16.58941	1.257045	0.4605126
5225	ENSBTAG00000013178	1.8233097	3.2171654	6.831173	0.0089580	80.43279	1.845853	0.6764641
8566	ENSBTAG00000021807	2.1697006	5.6998375	8.862451	0.0029110	455.80019	2.196048	0.7096633

Annotate the DEGs

```
annotation_ensembl_biomart<-getBM(attributes = c("ensembl_gene_id","hgnc_symbol",
                                                 "external_gene_name", "gene_biotype",
                                                 "description"), filters = "ensembl_gene_id",
                                  values = unique(stationB_merged_res_not_preg_preg_AI_sig$Row.names),
                                  mart = mart_bov)

stationB_merged_res_not_preg_preg_AI_sig_annotated<-
  merge(stationB_merged_res_not_preg_preg_AI_sig,annotation_ensembl_biomart, by.x="Row.names",
        by.y="ensembl_gene_id")
```

Row.names	logFC	logCPM	LR	PValue	baseMean	log2FoldChange	lfcSE	
ENSBTAG00000001308	1.9151902	3.6978275	6.895329	0.0086421	111.89445	1.899281	0.6933315	2.73[illegible]
ENSBTAG00000003414	-0.9760004	0.8983029	7.142139	0.0075293	14.43022	-1.009358	0.3187810	-3.16[illegible]
ENSBTAG00000012674	1.2764692	1.0871751	7.118853	0.0076277	16.58941	1.257045	0.4605126	2.72[illegible]
ENSBTAG00000013178	1.8233097	3.2171654	6.831173	0.0089580	80.43279	1.845853	0.6764641	2.72[illegible]
ENSBTAG00000021807	2.1697006	5.6998375	8.862451	0.0029110	455.80019	2.196048	0.7096633	3.09[illegible]

Calculating the eFDR

The calculation of the empirical FDR (eFDR) follows the principles and formula presented by Storey and Tibshirani (2003) PNAS, with a modification described in Sham and Purcell (2014) Nature Reviews Genetics.

The following code chunk was prepared for a multicore computer, and was not run here. Instead we upload the files and build figures produced from the files used for the paper.

```
permutation_matrix<-matrix(nrow=200000,ncol=12)

for (i in seq(1:200000)){
  sampling <- sample(1:12,12, replace = FALSE)
  if ( ! identical(sampling , c(1:12))){
    permutation_matrix[i,]<-sample(1:12,12, replace = FALSE)
  }}

permutation_matrix<-permutation_matrix[!duplicated(permutation_matrix),]
permutation_matrix<-permutation_matrix[sample(1:199963 , 10000),]
rand<-dim(permutation_matrix)[1]
sequence.pvalue<-seq(0.005, 0.05, 0.005)

results <- filebacked.big.matrix(length(sequence.pvalue),rand, type="double",
                                 init=0, separated=FALSE,
      backingfile="incidence_matrix.bin",
      descriptor="incidence_matrix.desc")
mdesc_result<- describe(results)

#calculate eFDR for edger

cl <- makeCluster(10)
registerDoParallel(cl)

results[,]<-foreach(i = sequence.pvalue, .combine='rbind', .inorder=TRUE,
```

```r
                    .packages=c("edgeR","bigmemory"), .verbose=TRUE) %:%
  foreach(j = 1:rand, .combine='cbind', .inorder=FALSE,.packages=c("edgeR","bigmemory"),
          .verbose=TRUE ) %dopar% {
    group<-factor(c("preg_AI","not_preg","not_preg","not_preg","preg_AI","preg_AI",
                    "preg_AI","preg_AI","not_preg","preg_AI","not_preg","not_preg"),
                  levels=c("preg_AI","not_preg"))
    group<-group[permutation_matrix[j,]]
    group<-factor(group, levels=c("preg_AI","not_preg"))
    design <- model.matrix(~group)

    dds<-DGEList(count=count_unfiltered_stationA, group=group)
    keep<-rowSums(cpm(dds)>1) >=6
    dds<-dds[keep, keep.lib.sizes=FALSE]
    dds<-estimateDisp(dds, design, robust=TRUE)
    dds <- glmFit(dds, design)
    dds <- glmLRT(dds)
    length(which(topTags(dds,n=Inf)$table$PValue <= i))
    }

stopCluster(cl)

total.rand <- rand * dim(count_pwbc_pairend1)[1]

qvalue<-data.frame(raw.pvalue = sequence.pvalue,
                    e.pvalue.edgeR= (rowSums(results[,]+1))/(total.rand+1),
                  e.pvalue.edgeR.round= round((rowSums(results[,]+1))/(total.rand+1),
                                              4))


rm(results)
system("rm incidence_matrix.bin")
system("rm incidence_matrix.desc")

#calculate eFDR for DESeq2

results.rand <- filebacked.big.matrix(length(sequence.pvalue),rand, type="double", init=0,
                                     separated=FALSE,
                                 backingfile="incidence_matrix.bin",
                                 descriptor="incidence_matrix.desc")
mdesc_result<- describe(results.rand)

cl <- makeCluster(10)
registerDoParallel(cl)

results.rand[,]<-foreach(i = sequence.pvalue, .combine='rbind', .inorder=TRUE,
                        .packages=c("DESeq2","bigmemory"), .verbose=TRUE) %:%

  foreach(j = 1:rand, .combine='cbind', .inorder=FALSE,.packages=c("DESeq2","bigmemory"),
          .verbose=TRUE ) %dopar% {

    group<-factor(c("preg_AI","not_preg","not_preg","not_preg","preg_AI","preg_AI","preg_AI",
                    "preg_AI","not_preg","preg_AI","not_preg","not_preg"),
                  levels=c("preg_AI","not_preg"))
```

```
    group<-group[permutation_matrix[j,]]
    group<-factor(group, levels=c("preg_AI","not_preg"))
    design <- model.matrix(~group)

   rownames(colData)<-colnames(count_unfiltered_stationA)
   count_filtered_stationA<-count_unfiltered_stationA[keep,]
   dds<-DESeqDataSetFromMatrix(countData=count_filtered_stationA, colData=colData, design= ~group)
   dds <- DESeq(dds)
   stationA_res_preg_bull_preg_AI_DESeq <- results(dds, contrast=c("group","not_preg" ,"preg_AI"),
                                                   pAdjustMethod="none", tidy=TRUE)
  length(which(stationA_res_preg_bull_preg_AI_DESeq$pvalue <= i))
  }

stopCluster(cl)

total.rand <- rand * dim(count_pwbc_pairend1)[1]

qvalue2<-data.frame(raw.pvalue = sequence.pvalue,
                   e.pvalue.DESEQ2= (rowSums(results.rand[,]+1))/(total.rand+1),
                   e.pvalue.DESEQ2.round= round((rowSums(results.rand[,]+1))/(total.rand+1)
                                                ,4))

qvalue3<-cbind(qvalue,qvalue2)

rm(results.rand)
system("rm incidence_matrix.bin")
system("rm incidence_matrix.desc")

write.table(qvalue3, file="2017_08_19_empirical_FDR_stationB.txt", quote=FALSE, sep="\t",
            row.names=FALSE)
```

Load the files with the results from the calculation of the empirical FDR to create the charts presented on Supplementary figure 1

```
eFDR_stationA<-read.delim('2017_08_19_empirical_FDR_stationA.txt.bz2',
                          row.names=1,header =TRUE,
                          stringsAsFactors =FALSE)
eFDR_stationB<-read.delim('2017_08_19_empirical_FDR_stationB.txt.bz2',
                          row.names=1,header =TRUE,
                          stringsAsFactors =FALSE)
```

```
plot1<-ggplot()+
  geom_point(data=eFDR_stationA, aes(x=e.pvalue.edgeR , y=raw.pvalue.1),
             color="black", size=1, shape=16)+
  geom_line(data=eFDR_stationA, aes(x=e.pvalue.edgeR , y=raw.pvalue.1),
            color="black", size=0.1,linetype=3)+
  geom_point(data=eFDR_stationA, aes(x=e.pvalue.DESEQ2 , y=raw.pvalue.1),
             color="black", size=1, shape=17)+
  geom_line(data=eFDR_stationA, aes(x=e.pvalue.DESEQ2 , y=raw.pvalue.1),
            color="black", size=0.1,linetype=3)+
  scale_y_continuous(name="nominal P", limits = c(0, 0.05), breaks=seq(0,0.05, 0.005))+
  scale_x_continuous(name="empirical eFDR", limits = c(0, 0.09), breaks=seq(0,0.09, 0.01))+
  ggtitle("Station A")+
  theme_bw()+
```

```
  theme(panel.grid= element_blank(),
        panel.background = element_blank(),
        panel.grid.minor = element_blank(),
        panel.grid.major = element_line(),
        plot.background = element_blank(),
        axis.title=element_text(color="black", size=8),
        axis.text=element_text(color="black", size=8),
        panel.spacing = unit(c(0.4,0.4,0.4,0.4),"cm"),
        plot.margin = unit(c(0.5,0.5,0.5,0.5),"cm"),
        legend.position="none",
        plot.title = element_text(lineheight=.8, hjust=0.5))

plot2<-ggplot()+
  geom_point(data=eFDR_stationB, aes(x=e.pvalue.edgeR , y=raw.pvalue.1),
             color="black", size=1, shape=16)+
  geom_line(data=eFDR_stationB, aes(x=e.pvalue.edgeR , y=raw.pvalue.1),
            color="black", size=0.1,linetype=3)+
  geom_point(data=eFDR_stationB, aes(x=e.pvalue.DESEQ2 , y=raw.pvalue.1),
             color="black", size=1, shape=17)+
  geom_line(data=eFDR_stationB, aes(x=e.pvalue.DESEQ2 , y=raw.pvalue.1),
            color="black", size=0.1,linetype=3)+
  scale_y_continuous(name="nominal P", limits = c(0, 0.05), breaks=seq(0,0.05, 0.005))+
  scale_x_continuous(name="empirical eFDR", limits = c(0, 0.09), breaks=seq(0,0.09, 0.01))+
  ggtitle("Station B")+
  theme_bw()+
  theme(panel.grid= element_blank(),
        panel.background = element_blank(),
        panel.grid.minor = element_blank(),
        panel.grid.major = element_line(),
        plot.background = element_blank(),
        axis.title=element_text(color="black", size=8),
        axis.text=element_text(color="black", size=8),
        panel.spacing = unit(c(0.4,0.4,0.4,0.4),"cm"),
        plot.margin = unit(c(0.5,0.5,0.5,0.5),"cm"),
        legend.position="none",
        plot.title = element_text(lineheight=.8, hjust=0.5))


multiplot(plot1,plot2, cols = 2)
```

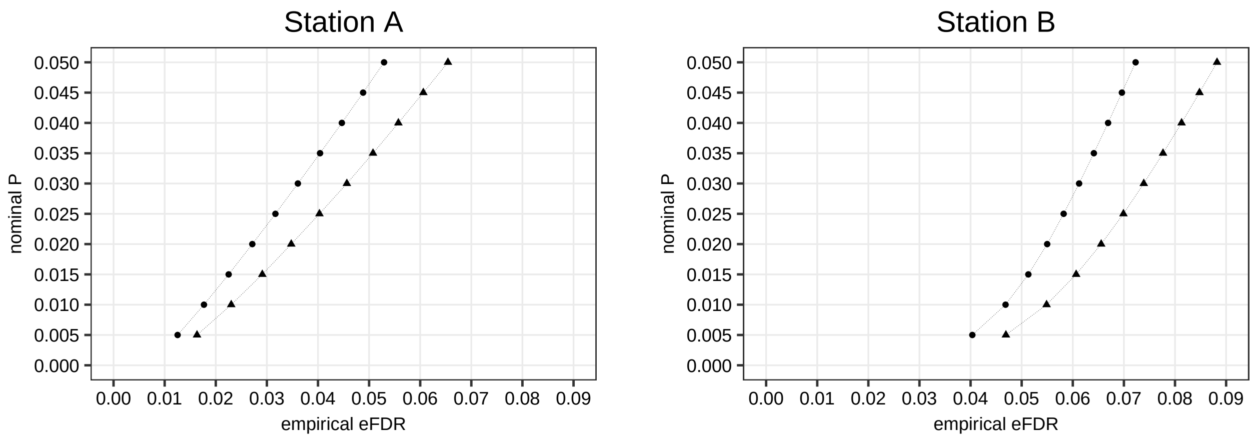

Results from edgeR were plotted with dots and DESeq2 were plotted with triangles

Pannel Figure 2

Fig 2a

```
station_A<-rownames(count_filtered_stationA)
station_B<-rownames(count_filtered_stationB)
```

Number of genes expressed in station A

```
length(station_A)
```

```
## [1] 12128
```

Number of genes expressed in station B

```
length(station_B)
```

```
## [1] 10832
```

Number of genes expressed in all samples

```
length(unique(c(station_A,station_B)))
```

```
## [1] 12538
```

```
ven_diagramm<-venn.diagram(list("station A" = station_A, "station B" = station_B),
             filename=NULL, euler.d=FALSE,scaled = FALSE,height = 700, width = 700,
             cat.cex=c(1,1),cex=1, cat.pos=c(3,3),
             cat.dist=c(0.1,0.1), hyper.test = FALSE)
```

```
grid.draw(ven_diagramm)
```

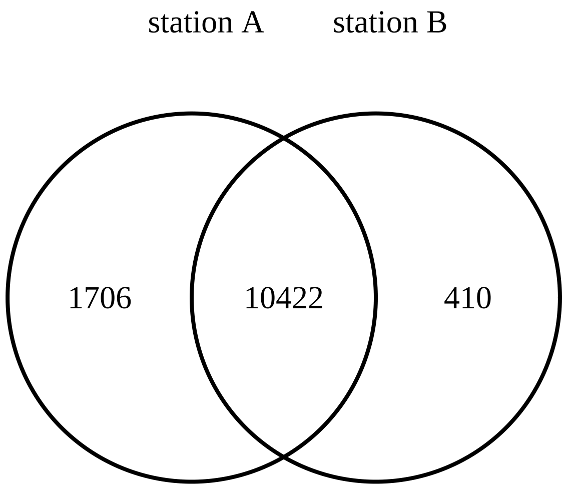

Fig 2b,c

```
stationA_merged_res_preg_bull_preg_AI$sig<-
  ifelse((stationA_merged_res_preg_bull_preg_AI$PValue <=0.01 &
            stationA_merged_res_preg_bull_preg_AI$pvalue <=0.01), "sig", "not_sig")
```

```r
stationA_merged_res_preg_bull_preg_AI_notsignificant<-
  stationA_merged_res_preg_bull_preg_AI[stationA_merged_res_preg_bull_preg_AI$sig=="not_sig",]
stationA_merged_res_preg_bull_preg_AI_significant<-
  stationA_merged_res_preg_bull_preg_AI[stationA_merged_res_preg_bull_preg_AI$sig=="sig",]
```

```r
stationB_merged_res_not_preg_preg_AI$sig<-
  ifelse((stationB_merged_res_not_preg_preg_AI$PValue <=0.01 &
          stationB_merged_res_not_preg_preg_AI$pvalue <=0.01), "sig", "not_sig")
stationB_merged_res_not_preg_preg_AI_notsignificant<-
  stationB_merged_res_not_preg_preg_AI[stationB_merged_res_not_preg_preg_AI$sig=="not_sig",]
stationB_merged_res_not_preg_preg_AI_significant<-
  stationB_merged_res_not_preg_preg_AI[stationB_merged_res_not_preg_preg_AI$sig=="sig",]
```

```r
plot1<-ggplot()+
  geom_hline(yintercept = 0, color="grey")+
  geom_vline(xintercept = 0, color="grey")+
  geom_point(data=stationA_merged_res_preg_bull_preg_AI_notsignificant,
             aes(x=logFC , y=log2FoldChange), color="black", size=1, shape=16, alpha=0.8)+
  geom_point(data=stationA_merged_res_preg_bull_preg_AI_significant,
             aes(x=logFC , y=log2FoldChange), fill="gray",color="red",size=2, shape = 21)+
  scale_y_continuous(name="DESeq2 \n Log2[FC(Pregnant NB/Pregnant AI)]", limits = c(-4.2, 4.2))+
  scale_x_continuous(name="Log2[FC(Pregnant NB/Pregnant AI)] \n edgeR", limits = c(-4.2, 4.2))+
  ggtitle("Station A")+
  theme_bw()+
  theme(panel.grid= element_blank(),
        panel.background = element_blank(),
        panel.grid.minor = element_blank(),
        panel.grid.major = element_blank(),
        plot.background = element_blank(),
        aspect.ratio =1,
        axis.title=element_text(color="black", size=10),
        axis.text=element_text(color="black", size=14),
        panel.spacing = unit(c(0.4,0.4,0.4,0.4),"cm"),
        plot.margin = unit(c(0.5,0.5,0.5,0.5),"cm"),
        legend.position="none",
        plot.title = element_text(lineheight=.8, hjust=0.5))

plot2<-ggplot()+
  geom_hline(yintercept = 0, color="grey")+
  geom_vline(xintercept = 0, color="grey")+
  geom_point(data=stationB_merged_res_not_preg_preg_AI_notsignificant,
             aes(x=logFC , y=log2FoldChange), color="black", size=1, shape=16, alpha=0.8)+
  geom_point(data=stationB_merged_res_not_preg_preg_AI_significant,
             aes(x=logFC , y=log2FoldChange), fill="gray",color="red",size=2, shape = 21)+
  scale_y_continuous(name="DESeq2 \n Log2[FC(Not Pregnant/Pregnant AI)]", limits = c(-4, 4))+
  scale_x_continuous(name="Log2[FC(Not Pregnant/Pregnant AI)] \n edgeR", limits = c(-4, 4))+
  theme_bw()+
  ggtitle("Station B")+
  theme(panel.grid= element_blank(),
        panel.background = element_blank(),
        panel.grid.minor = element_blank(),
        panel.grid.major = element_blank(),
        plot.background = element_blank(),
        aspect.ratio =1,
```

```
        axis.title=element_text(color="black", size=10),
        axis.text=element_text(color="black", size=14),
        panel.spacing = unit(c(0.4,0.4,0.4,0.4),"cm"),
        plot.margin = unit(c(0.5,0.5,0.5,0.5),"cm"),
        legend.position="none",
        plot.title = element_text(lineheight=.8, hjust=0.5)
        )


multiplot(plot1,plot2, cols = 2)
```

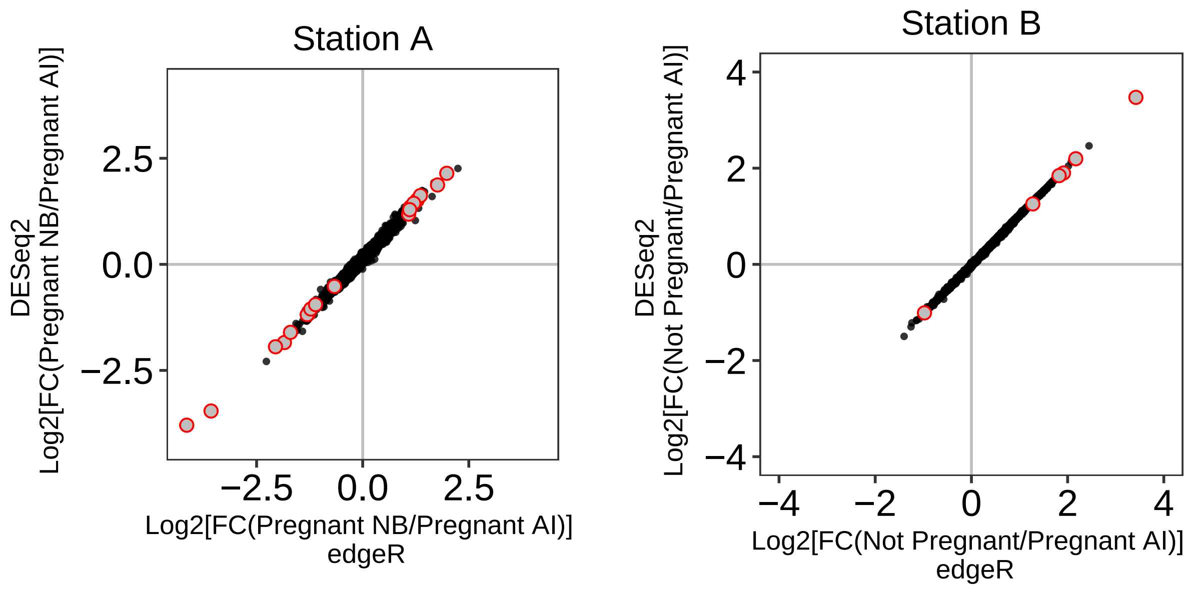

Fig 2d,e

All the DEGs were plot together to improve the charts' aesthetics. For the paper we split them into Fig 2d and Fig 2e.

```
#Station A
group<-factor(c("preg_AI","preg_AI","preg_AI","preg_bull","preg_bull","preg_bull",
                "preg_AI","preg_AI","preg_bull","preg_bull","preg_AI"),
              levels=c("preg_AI","preg_bull"))
dds<-DGEList(count=count_unfiltered_stationA, group=group)
keep<-rowSums(cpm(dds)>1) >=6
dds<-dds[keep , keep.lib.sizes=FALSE]
counts_per_million_stationA<-cpm(dds)

stationA_merged_res_preg_bull_preg_AI_sig_annotated_CPM<-
  merge(stationA_merged_res_preg_bull_preg_AI_sig_annotated,counts_per_million_stationA,
        by.x="Row.names", by.y="row.names", all.x=TRUE,all.y=FALSE)

data_chart2<-data.frame()
for (i in seq(1,dim(stationA_merged_res_preg_bull_preg_AI_sig_annotated_CPM)[1],1)){
  geneID<-rep(stationA_merged_res_preg_bull_preg_AI_sig_annotated_CPM[i,1],1)
  cpm<-stationA_merged_res_preg_bull_preg_AI_sig_annotated_CPM[i,c(16:26)]
  data_chart<-data.frame(cbind(geneID, t(cpm)[,1]),stringsAsFactors=FALSE)
```

```r
  data_chart$chart<-i
  data_chart$shape<-c(0:10)
  colnames(data_chart)<-c("geneID", "cpm", "chart", "shape")
  data_chart2<-rbind(data_chart2,data_chart)
}

data_chart2$cpm<-as.numeric(data_chart2$cpm)

data_chart2$group<-rep(group,18)

annotation_ensembl_biomart<-getBM(attributes = c("ensembl_gene_id","external_gene_name"),
                                  filters = "ensembl_gene_id",
                                  values = unique(data_chart2$geneID), mart = mart_bov)

annotation_ensembl_biomart$external_gene_name<-
  ifelse(annotation_ensembl_biomart$external_gene_name == '',
         annotation_ensembl_biomart$ensembl_gene_id,
         annotation_ensembl_biomart$external_gene_name )

data_chart2<-merge(data_chart2, annotation_ensembl_biomart, by.x="geneID", by.y="ensembl_gene_id")

data_chart2$external_gene_name[data_chart2$geneID=='ENSBTAG00000021077']<-'BOLA-DQB'
data_chart2$external_gene_name[data_chart2$geneID=='ENSBTAG00000045492']<-'ANG'
data_chart2$external_gene_name[data_chart2$geneID=='ENSBTAG00000047764']<-'LOC107131247'
data_chart2$external_gene_name[data_chart2$geneID=='ENSBTAG00000035868']<-'SIGLEC14'
data_chart2$external_gene_name[data_chart2$geneID=='ENSBTAG00000040580']<-'LOC618633'
data_chart2$external_gene_name[data_chart2$geneID=='ENSBTAG00000022715']<-'DMBT1'

data_chart2$external_gene_name<-factor(data_chart2$external_gene_name,
                                       levels=c(sort(unique(data_chart2$external_gene_name))))
data_chart2_stationA<-data_chart2

rm(data_chart2,group)

#Station B

group<-factor(c("preg_AI","not_preg","not_preg","not_preg","preg_AI","preg_AI",
                "preg_AI","preg_AI","not_preg","preg_AI","not_preg","not_preg"),
              levels=c("preg_AI","not_preg"))
dds<-DGEList(count=count_unfiltered_stationB, group=group)
keep<-rowSums(cpm(dds)>1) >=6
dds<-dds[keep , keep.lib.sizes=FALSE]
counts_per_million_stationB<-cpm(dds)

stationB_merged_res_not_preg_preg_AI_sig_annotated_CPM<-
  merge(stationB_merged_res_not_preg_preg_AI_sig_annotated,counts_per_million_stationB,
        by.x="Row.names", by.y="row.names", all.x=TRUE,all.y=FALSE)

data_chart2<-data.frame()
for (i in seq(1,dim(stationB_merged_res_not_preg_preg_AI_sig_annotated_CPM)[1],1)){
  geneID<-rep(stationB_merged_res_not_preg_preg_AI_sig_annotated_CPM[i,1],1)
  cpm<-stationB_merged_res_not_preg_preg_AI_sig_annotated_CPM[i,c(16:27)]
  data_chart<-data.frame(cbind(geneID, t(cpm)[,1]),stringsAsFactors=FALSE)
```

```r
  data_chart$chart<-i
  data_chart$shape<-c(0:10,12)
  colnames(data_chart)<-c("geneID", "cpm", "chart", "shape")
  data_chart2<-rbind(data_chart2,data_chart)
}

data_chart2$cpm<-as.numeric(data_chart2$cpm)

data_chart2$group<-rep(group,6)

annotation_ensembl_biomart<-getBM(attributes = c("ensembl_gene_id","external_gene_name"),
                                  filters = "ensembl_gene_id",
                                  values = unique(data_chart2$geneID), mart = mart_bov)

annotation_ensembl_biomart$external_gene_name<-
  ifelse(annotation_ensembl_biomart$external_gene_name == '',
         annotation_ensembl_biomart$ensembl_gene_id,
         annotation_ensembl_biomart$external_gene_name )


data_chart2<-merge(data_chart2, annotation_ensembl_biomart, by.x="geneID", by.y="ensembl_gene_id")

data_chart2$external_gene_name[data_chart2$external_gene_name=="ENSBTAG00000001308"]<-"LOC522763"

data_chart2$external_gene_name<-factor(data_chart2$external_gene_name,
                                       levels=c(sort(unique(data_chart2$external_gene_name))))

data_chart2_stationB<-data_chart2

data_chart_joint<-rbind(data_chart2_stationA,data_chart2_stationB)
```

```r
ggplot(data=data_chart_joint, aes(x=group , y=cpm, colour=group))+
  geom_jitter(position = position_jitter(width = .3, height=0), size=2,
             shape=as.factor(data_chart_joint$shape))+
  scale_x_discrete("",labels=c("preg_AI"="Pregnant AI", "preg_bull"="Pregnant NB", "not_preg"="Not Pregn
  scale_colour_manual(name=NULL, values = c("blue", "darkgreen","red"))+
  stat_summary(fun.y = median, fun.ymin = median, fun.ymax = median,
              colour = "gray", size = 0.3,  geom = "crossbar", alpha=0.5)+
  scale_y_continuous("CPM")+
  facet_wrap(~ external_gene_name , scales="free_y", nrow=3, ncol=9)+
  theme_bw()+
  theme(panel.grid= element_blank(),
        panel.background = element_blank(),
        panel.grid.minor = element_blank(),
        panel.grid.major = element_blank(),
        plot.background = element_blank(),
        strip.background = element_rect(fill = "white"),
        strip.text.x = element_text(colour = 'black', face="italic",size = 9),
        legend.text = element_text( colour = 'black', size = 10 ),
        axis.text.x = element_blank(),
        axis.text.y = element_text( colour = 'black', size = 10 ),
        legend.key.size = unit(0.9,"cm"),
        legend.position="none",
        axis.ticks.x = element_blank()
```

```
)
```

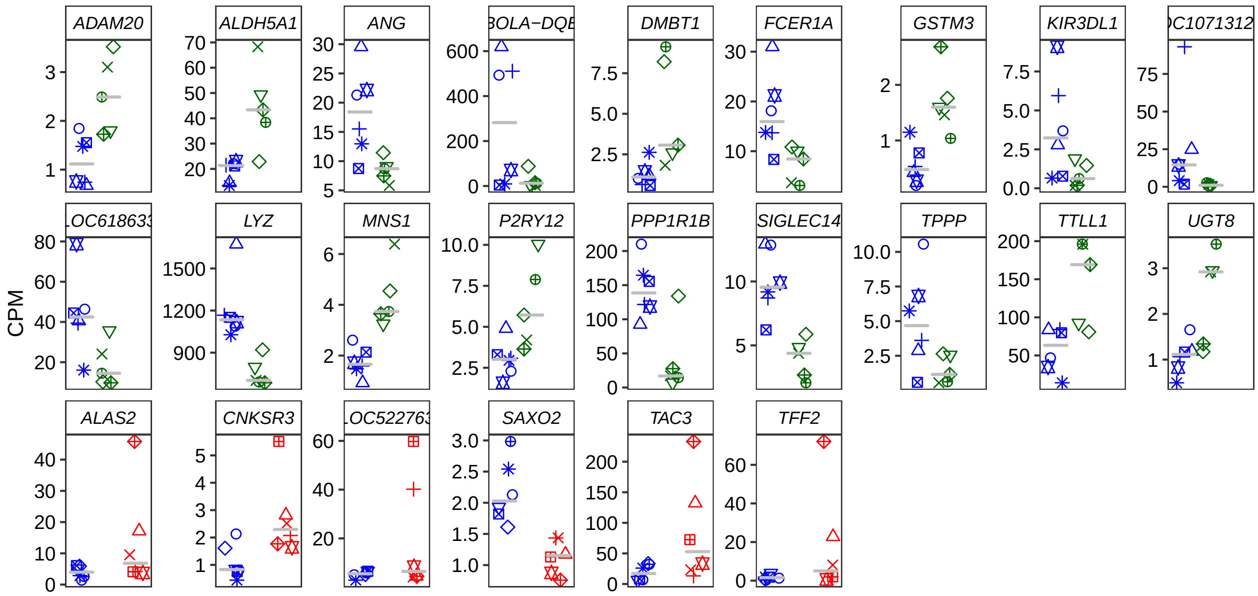

TSP for STATION A

Load the filtered FPKM data

```
FPKM_stationA<-read.delim('2017_09_07_FPKM_filtered_station_A.txt.bz2',
                          row.names=1,header =TRUE,stringsAsFactors =FALSE)
```

	SL220764	SL220765	SL220766	SL220767	SL220768	SL220769	SL220771	SL2207
ENSBTAG00000000005	46.490682	14.046891	40.627510	7.6508059	47.895741	51.426536	8.3066798	10.1704
ENSBTAG00000000010	56.653441	68.073498	56.241052	89.1360136	49.681829	53.963357	57.4911046	85.3813
ENSBTAG00000000012	13.635656	10.225421	14.166677	6.4219761	13.679317	14.596885	15.8842118	9.7979
ENSBTAG00000000013	9.500259	6.858602	10.505388	6.5143390	11.042161	10.683956	7.6669004	7.0289
ENSBTAG00000000014	88.120425	98.635591	96.096566	78.9679034	74.162299	87.390160	67.1902158	71.7568
ENSBTAG00000000015	4.706462	1.663422	4.389125	0.4384998	6.059784	6.458674	0.5596494	1.3398

Calculate the top scoring pairs (TSPs).

```
group<-c("preg_AI","preg_AI","preg_AI","preg_bull","preg_bull",
         "preg_bull","preg_AI","preg_AI","preg_bull","preg_bull",
         "preg_AI")
tsp_stationA <- tspcalc(as.matrix(FPKM_stationA),group)
#out <- tspsig(as.matrix(FPKM_stationA),group,B=5000,seed=12355)
# did not run the calculation for p-value because it takes hours to complete
```

TSP for STATION B

Load the filtered FPKM data

```
FPKM_stationB<-read.delim('2017_09_07_FPKM_filtered_station_B.txt.bz2' ,
                          row.names=1,header =TRUE,stringsAsFactors =FALSE)
```

	SL253803	SL253804	SL253805	SL253806	SL253807	SL253808	SL253809	SL253810
ENSBTAG00000000005	44.464538	40.586976	38.983254	30.97536	46.67648	43.06311	33.966120	37.147074
ENSBTAG00000000010	13.179562	52.254752	9.777053	14.64029	12.44155	58.15886	13.381804	17.269678
ENSBTAG00000000012	34.259276	16.773969	27.916959	32.91785	36.18264	15.78470	34.989841	30.828511
ENSBTAG00000000013	34.895103	13.831557	26.702725	27.20706	30.39308	11.54701	33.337640	20.742905
ENSBTAG00000000014	8.301100	84.705340	8.583813	15.02244	13.10523	78.47710	11.371698	24.113591
ENSBTAG00000000016	5.810031	9.237036	1.010546	2.27675	3.14642	10.22144	3.964776	2.801302

Calculate the top scoring pairs (TSPs).

```
group<-c("preg_AI","not_preg","not_preg","not_preg","preg_AI","preg_AI",
         "preg_AI","preg_AI","not_preg","preg_AI","not_preg","not_preg")
tsp_stationB <- tspcalc(as.matrix(FPKM_stationB),group)
#out <- tspsig(as.matrix(FPKM_stationB),group,B=5000,seed=12355)
#did not run the calculation for p-value because it takes hours to complete
```

TSP for STATIONS A & B

Note that for the calculation of TSP for stations A and B, we separated the samples into the binary categories "preg_AI","not_preg_AI".

```
FPKM_stationAandB<-merge(FPKM_stationA,FPKM_stationB, by='row.names', all=FALSE)
rownames(FPKM_stationAandB)<-FPKM_stationAandB$Row.names
FPKM_stationAandB<-FPKM_stationAandB[,2:24]
group<-c("preg_AI","preg_AI","preg_AI","not_preg_AI","not_preg_AI",
         "not_preg_AI","preg_AI","preg_AI","not_preg_AI","not_preg_AI",
         "preg_AI","preg_AI","not_preg_AI","not_preg_AI","not_preg_AI",
         "preg_AI","preg_AI","preg_AI","preg_AI","not_preg_AI","preg_AI",
         "not_preg_AI","not_preg_AI")

tsp_stationAandB <- tspcalc(as.matrix(FPKM_stationAandB),group)
#out <- tspsig(as.matrix(FPKM_stationAandB),group,B=10000,seed=12355)
```

Pannel Figure 3

Fig 3a

The top panel of Fig 3a is the first graph (top,left) of the scatterplots for the top 20 TSPs (Supplementary Figure 2a).

```
annotation_ensembl_biomart<-getBM(attributes = c("ensembl_gene_id","external_gene_name"),
                                  values="*", mart = mart_bov)
```

```r
tsp_stationA_index<-as.data.frame(tsp_stationA$index)
tsp_stationA_index[,3]<-tsp_stationA$score
tsp_stationA_index[,4]<-tsp_stationA$tspscore
tsp_stationA_index<-tsp_stationA_index[with(tsp_stationA_index, order(-V3)), ]
tsp_stationA_index<-as.data.frame(tsp_stationA_index)

group<-c("preg_AI","preg_AI","preg_AI","preg_bull","preg_bull",
         "preg_bull","preg_AI","preg_AI","preg_bull","preg_bull",
         "preg_AI")

data_for_TSP_chart<-data.frame()
data_for_TSP_chart_multiple<-data.frame()
for (i in c(1:20)) {
  data_for_TSP_chart<-FPKM_stationA[c(tsp_stationA_index[i,1],tsp_stationA_index[i,2]),]
  gene_1<-rownames(data_for_TSP_chart)[1]
  gene_2<-rownames(data_for_TSP_chart)[2]
  data_for_TSP_chart<- data.frame(t( data_for_TSP_chart ))
  data_for_TSP_chart$group<-factor(group, levels=c("preg_AI", "preg_bull"))
  data_for_TSP_chart$chart<-i
  data_for_TSP_chart$gene_1<-gene_1
  data_for_TSP_chart$gene_2<-gene_2
  colnames(data_for_TSP_chart)<-c("gene_1_fpkm","gene_2_fpkm","group","chart","gene_1","gene_2")
  data_for_TSP_chart_multiple<-rbind(data_for_TSP_chart_multiple,data_for_TSP_chart)
}

data_for_TSP_chart_multiple$gene_1_symbol<-
  annotation_ensembl_biomart$external_gene_name[match(data_for_TSP_chart_multiple$gene_1,
                                                      annotation_ensembl_biomart$ensembl_gene_id)]
data_for_TSP_chart_multiple$gene_2_symbol<-
  annotation_ensembl_biomart$external_gene_name[match(data_for_TSP_chart_multiple$gene_2,
                                                      annotation_ensembl_biomart$ensembl_gene_id)]

data_for_TSP_chart_multiple$gene_1_symbol<-
  ifelse(!(data_for_TSP_chart_multiple$gene_1_symbol==""), data_for_TSP_chart_multiple$gene_1_symbol,
         data_for_TSP_chart_multiple$gene_1)
data_for_TSP_chart_multiple$gene_2_symbol<-
  ifelse(!(data_for_TSP_chart_multiple$gene_2_symbol==""), data_for_TSP_chart_multiple$gene_2_symbol,
         data_for_TSP_chart_multiple$gene_2)
```

```r
plots <- list()
for (i  in c(1:20)){
data_for_TSP_chart_multiple_a<-data_for_TSP_chart_multiple[data_for_TSP_chart_multiple$chart %in% i,]
plot<-ggplot(data=data_for_TSP_chart_multiple_a, aes(x=gene_1_fpkm,y=gene_2_fpkm))+
  geom_point(aes(colour=group),size=0.4)+
  scale_color_manual(values=c("blue","green"),name="Group",labels=c("Preg AI", "preg_bull"))+
  geom_abline(intercept = 0, slope = 1, color="gray",size=0.2)+
  scale_y_continuous(name=data_for_TSP_chart_multiple_a$gene_2_symbol[1] ,
                     limits=c(min(data_for_TSP_chart_multiple_a$gene_1_fpkm,
                                  data_for_TSP_chart_multiple_a$gene_2_fpkm),
                              max(data_for_TSP_chart_multiple_a$gene_1_fpkm,
                                  data_for_TSP_chart_multiple_a$gene_2_fpkm)))+
  scale_x_continuous(name=data_for_TSP_chart_multiple_a$gene_1_symbol[1],
                     limits = c(min(data_for_TSP_chart_multiple_a$gene_1_fpkm,
                                    data_for_TSP_chart_multiple_a$gene_2_fpkm),
```

```
                                 max(data_for_TSP_chart_multiple_a$gene_1_fpkm,
                                     data_for_TSP_chart_multiple_a$gene_2_fpkm)))+
  theme(aspect.ratio = 1,
        panel.grid.major = element_blank(),
        panel.grid.minor = element_blank(),
        panel.background = element_rect(fill="gray96"),
        plot.background  = element_blank(),
        axis.text.x = element_text( colour = 'black' ,size = 7),
        axis.text.y = element_text( colour = 'black',size = 7),
        axis.title= element_text( colour = 'black' ,size = 7, face="italic"),
        axis.ticks = element_line(size=0.1),
        panel.spacing = unit(1, "mm"),
        legend.position="none")
plots[[i]] <- plot

}
multiplot(plotlist = plots, cols = 5,layout = matrix(1:20, nrow=4, byrow = TRUE))
```

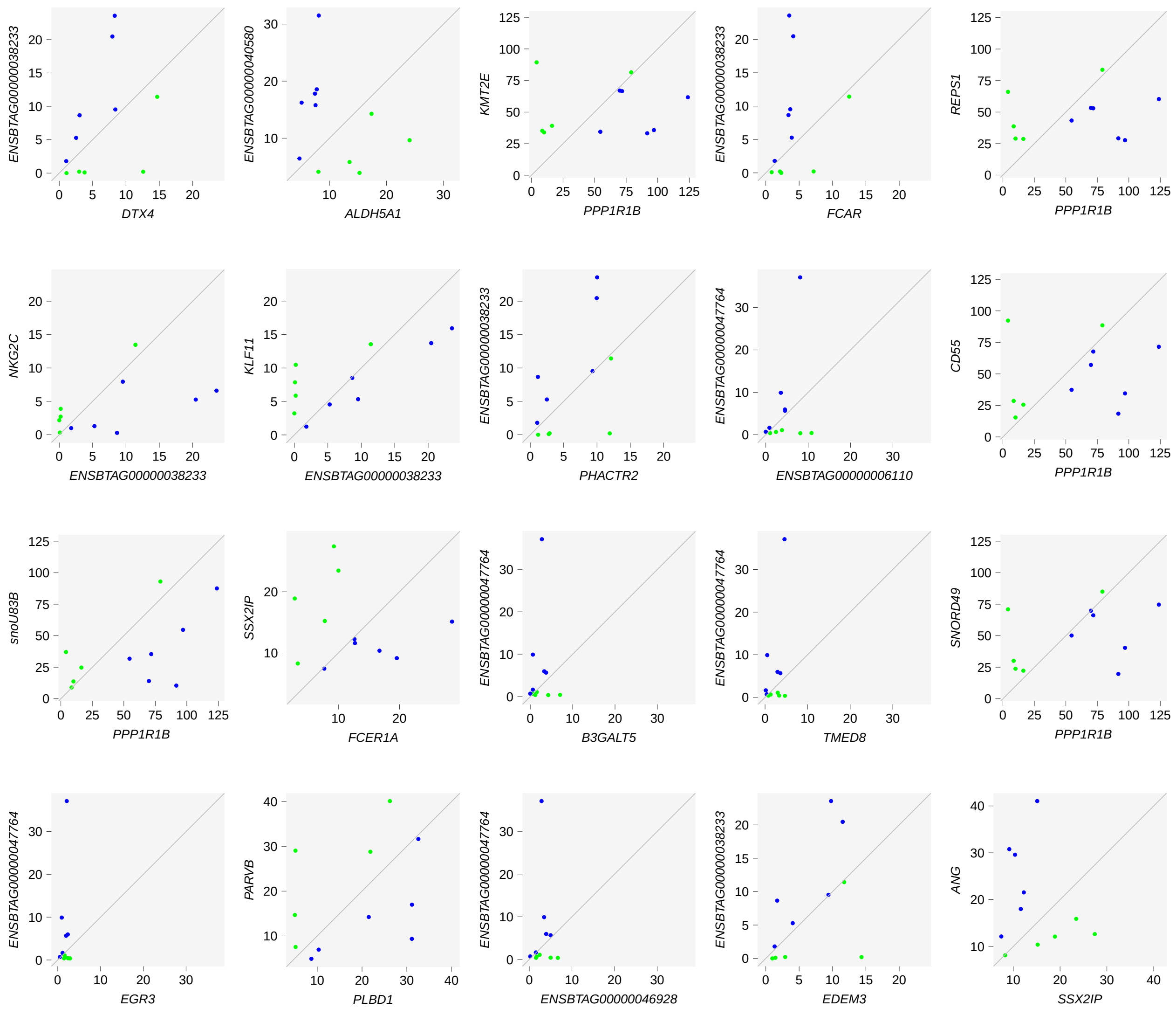

Fig 3b

First, we calculated the clusters, and the certainty of their formation, using the ratio of gene expression for the top 20 TSPs.

```
data_for_TSP_chart<-data.frame()
data_for_TSP_chart_multiple<-data.frame()
for (i in c(1:20)) {
  data_for_TSP_chart<-FPKM_stationA[c(tsp_stationA_index[i,1],tsp_stationA_index[i,2]),]
  gene_1<-rownames(data_for_TSP_chart)[1]
  gene_2<-rownames(data_for_TSP_chart)[2]
  data_for_TSP_chart<- data.frame(t( data_for_TSP_chart ))
  data_for_TSP_chart$group<-factor(group, levels=c("preg_AI", "preg_bull"))
  data_for_TSP_chart$samples<-rownames(data_for_TSP_chart)
  data_for_TSP_chart<-data_for_TSP_chart[with(data_for_TSP_chart, order(group,samples)), ]

   if(data_for_TSP_chart[1,1] > data_for_TSP_chart[1,2]) {

    data_for_TSP_chart$ratio<-log2(data_for_TSP_chart[,1]+1) - log2(data_for_TSP_chart[,2]+1)

    data_for_TSP_chart$gene_1<-gene_1
    data_for_TSP_chart$gene_2<-gene_2

  } else{data_for_TSP_chart$ratio<-log2(data_for_TSP_chart[,2]+1) - log2(data_for_TSP_chart[,1]+1)
  data_for_TSP_chart$gene_1<-gene_2
  data_for_TSP_chart$gene_2<-gene_1
  }

  colnames(data_for_TSP_chart)<-c("gene1", "gene2", "group", "samples", "ratio", "gene_1", "gene_2" )
  data_for_TSP_chart_multiple<-rbind(data_for_TSP_chart_multiple,data_for_TSP_chart)
}
head(data_for_TSP_chart_multiple)


data_for_TSP_heatmap <- dcast(data_for_TSP_chart_multiple,
                              formula = gene_1 + gene_2 ~ samples, value.var = "ratio")
head(data_for_TSP_heatmap)
dim(data_for_TSP_heatmap)
data_for_TSP_heatmap$gene_1_symbol<-
  annotation_ensembl_biomart$external_gene_name[match(data_for_TSP_heatmap$gene_1,
                                                      annotation_ensembl_biomart$ensembl_gene_id)]
data_for_TSP_heatmap$gene_2_symbol<-
  annotation_ensembl_biomart$external_gene_name[match(data_for_TSP_heatmap$gene_2,
                                                      annotation_ensembl_biomart$ensembl_gene_id)]
data_for_TSP_heatmap$gene_1_symbol<-
  ifelse(!(data_for_TSP_heatmap$gene_1_symbol==""), data_for_TSP_heatmap$gene_1_symbol,
         data_for_TSP_heatmap$gene_1)
data_for_TSP_heatmap$gene_2_symbol<-
  ifelse(!(data_for_TSP_heatmap$gene_2_symbol==""), data_for_TSP_heatmap$gene_2_symbol,
         data_for_TSP_heatmap$gene_2)
data_for_TSP_heatmap$gene_order<-paste(data_for_TSP_heatmap$gene_1_symbol, ">",
                                       data_for_TSP_heatmap$gene_2_symbol, sep=" ")
```

```r
result<-pvclust(data_for_TSP_heatmap[,c(3:13)], method.dist="euclidean",
                method.hclust="average",nboot=5000, parallel=FALSE)
plot(result)
```

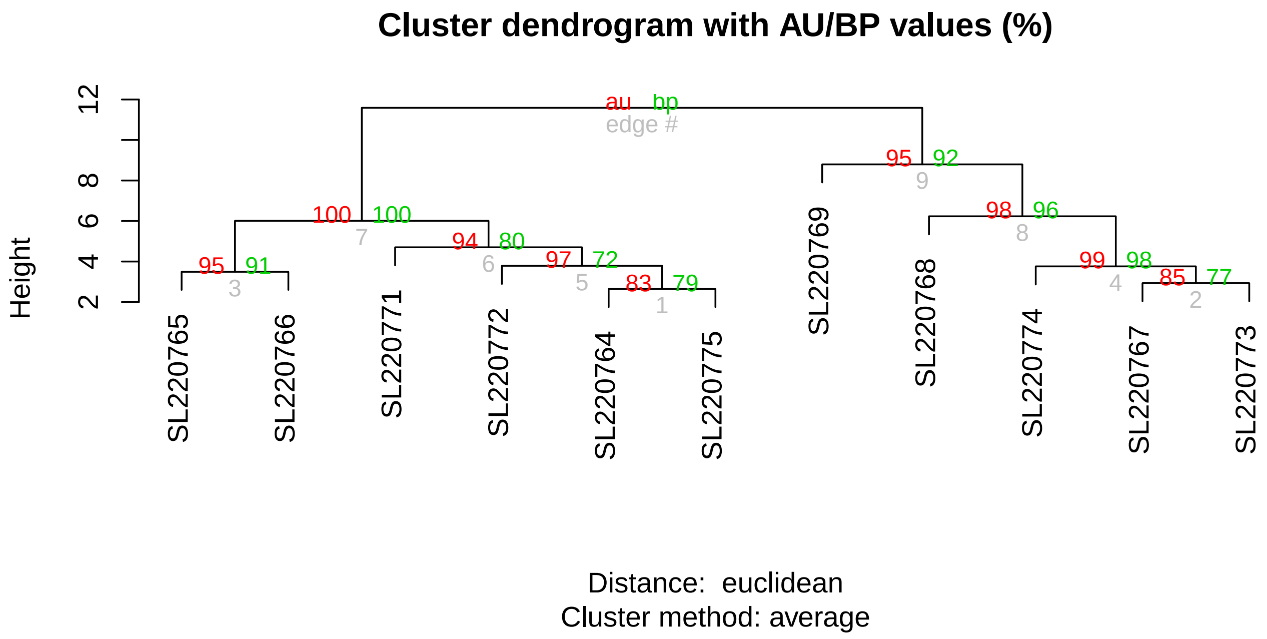

Next we plotted the heatmap.

```r
row_annotation<-data_for_TSP_heatmap$gene_order
row_annotation<-row_annotation[hclust(dist(data_for_TSP_heatmap[,c(3:13)],
                                          method = "manhattan"),method = "complete")$order]
draw(
Heatmap(data_for_TSP_heatmap[,c(3:13)],
        name = "Log2(Gene1)-Log2(Gene2)",
        cluster_rows= hclust(dist(data_for_TSP_heatmap[,c(3:13)],
                                  method = "manhattan"),method = "complete"),
        cluster_columns= color_branches( result$hclust , k=2, col=c("blue","green") ),
        show_column_names = TRUE,
        show_row_dend = FALSE,
        col = colorRamp2(c(-3, 0, 3), c("red", "white", "blue")),
        heatmap_legend_param = list(color_bar = "continuous",
                                    legend_direction = "horizontal",
                                    title_position = "lefttop",grid_height = unit(2, "mm"),
                                    gap=unit(1,"mm"),labels_gp = gpar(fontsize = 7),
                                    title_gp = gpar(fontsize = 7))) +

        rowAnnotation(link = row_anno_link(at= hclust(dist(data_for_TSP_heatmap[,c(3:13)],
                                          method = "manhattan"),method = "complete")$order,
                                          labels = row_annotation,
                                          labels_gp = gpar(fontsize = 8) ),
                      width = unit(2, "mm") +
        max_text_width(row_annotation, gp = gpar(fontsize = 8)))
        ,heatmap_legend_side = "bottom"
)
```

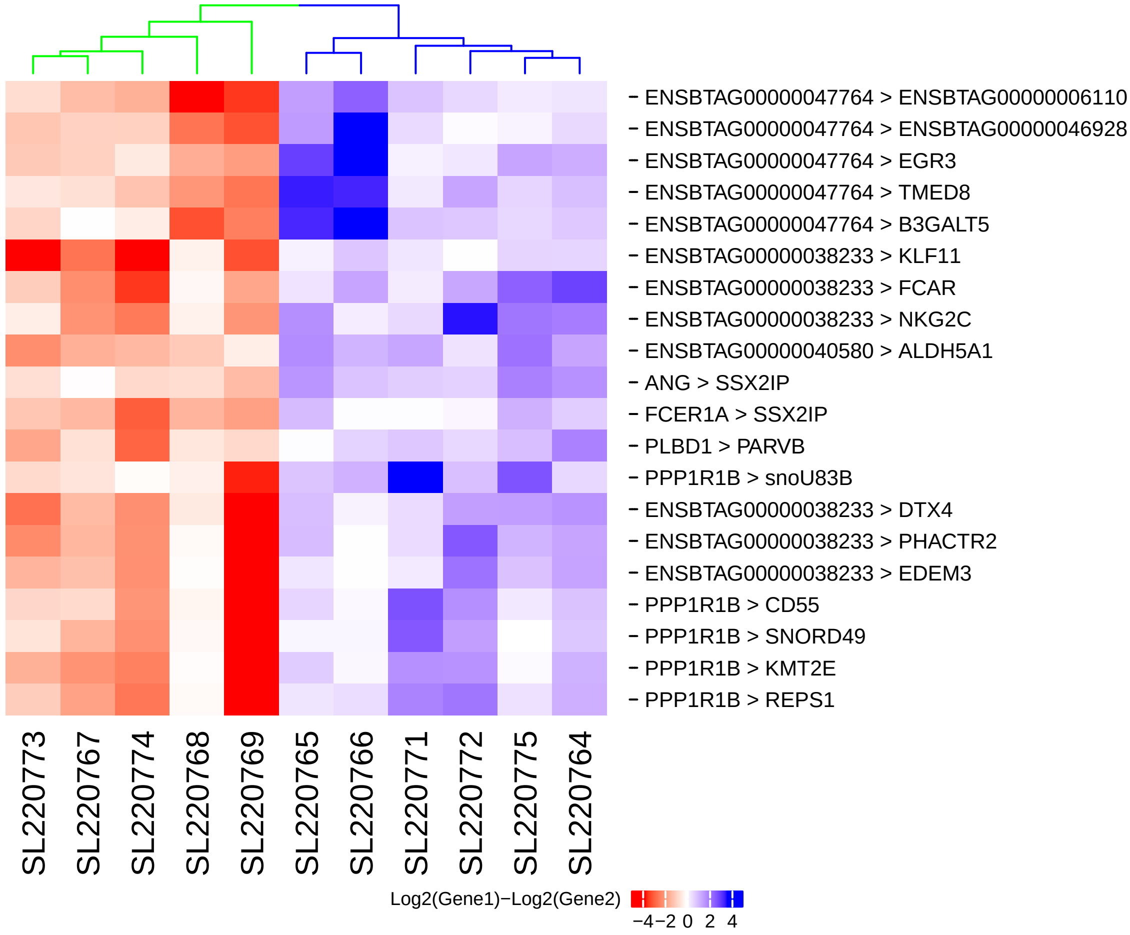

Fig 3c

The top panel of Fig 3c is the first graph (top,left) of the scatterplots for the top 20 TSPs (Supplementary Figure 2b).

```
tsp_stationB_index<-as.data.frame(tsp_stationB$index)
tsp_stationB_index[,3]<-tsp_stationB$score
tsp_stationB_index[,4]<-tsp_stationB$tspscore
tsp_stationB_index<-tsp_stationB_index[with(tsp_stationB_index, order(-V3)), ]
tsp_stationB_index<-as.data.frame(tsp_stationB_index)

group<-c("preg_AI","not_preg","not_preg","not_preg","preg_AI","preg_AI","preg_AI",
         "preg_AI","not_preg","preg_AI","not_preg","not_preg")

data_for_TSP_chart<-data.frame()
data_for_TSP_chart_multiple<-data.frame()
for (i in c(1:20)) {
  data_for_TSP_chart<-FPKM_stationB[c(tsp_stationB_index[i,1],tsp_stationB_index[i,2]),]
  gene_1<-rownames(data_for_TSP_chart)[1]
  gene_2<-rownames(data_for_TSP_chart)[2]
  data_for_TSP_chart<- data.frame(t( data_for_TSP_chart ))
  data_for_TSP_chart$group<-factor(group, levels=c("preg_AI", "not_preg"))
  data_for_TSP_chart$chart<-i
```

```
  data_for_TSP_chart$gene_1<-gene_1
  data_for_TSP_chart$gene_2<-gene_2
  colnames(data_for_TSP_chart)<-c("gene_1_fpkm","gene_2_fpkm","group","chart","gene_1","gene_2")
  data_for_TSP_chart_multiple<-rbind(data_for_TSP_chart_multiple,data_for_TSP_chart)
}

data_for_TSP_chart_multiple$gene_1_symbol<-
  annotation_ensembl_biomart$external_gene_name[match(data_for_TSP_chart_multiple$gene_1,
                                                      annotation_ensembl_biomart$ensembl_gene_id)]
data_for_TSP_chart_multiple$gene_2_symbol<-
  annotation_ensembl_biomart$external_gene_name[match(data_for_TSP_chart_multiple$gene_2,
                                                      annotation_ensembl_biomart$ensembl_gene_id)]

data_for_TSP_chart_multiple$gene_1_symbol<-
  ifelse(!(data_for_TSP_chart_multiple$gene_1_symbol==""), data_for_TSP_chart_multiple$gene_1_symbol,
         data_for_TSP_chart_multiple$gene_1)
data_for_TSP_chart_multiple$gene_2_symbol<-
  ifelse(!(data_for_TSP_chart_multiple$gene_2_symbol==""), data_for_TSP_chart_multiple$gene_2_symbol,
         data_for_TSP_chart_multiple$gene_2)
```

```
plots <- list()
for (i  in c(1:20)){
data_for_TSP_chart_multiple_a<-data_for_TSP_chart_multiple[data_for_TSP_chart_multiple$chart %in% i,]
plot<-ggplot(data=data_for_TSP_chart_multiple_a, aes(x=gene_1_fpkm,y=gene_2_fpkm))+
  geom_point(aes(colour=group),size=0.4)+
  scale_color_manual(values=c("blue","red"),name="Group",labels=c("Preg AI", "Not Preg"))+
  geom_abline(intercept = 0, slope = 1, color="gray",size=0.2)+
  scale_y_continuous(name=data_for_TSP_chart_multiple_a$gene_2_symbol[1] ,
                     limits = c(min(data_for_TSP_chart_multiple_a$gene_1_fpkm,
                                    data_for_TSP_chart_multiple_a$gene_2_fpkm),
                                max(data_for_TSP_chart_multiple_a$gene_1_fpkm,
                                    data_for_TSP_chart_multiple_a$gene_2_fpkm)))+
  scale_x_continuous(name=data_for_TSP_chart_multiple_a$gene_1_symbol[1],
                     limits = c(min(data_for_TSP_chart_multiple_a$gene_1_fpkm,
                                    data_for_TSP_chart_multiple_a$gene_2_fpkm),
                                max(data_for_TSP_chart_multiple_a$gene_1_fpkm,
                                    data_for_TSP_chart_multiple_a$gene_2_fpkm)))+
  #facet_wrap( ~ chart + gene_1_symbol + gene_2_symbol, nrow=2)+
  theme(aspect.ratio = 1,
        panel.grid.major = element_blank(),
        panel.grid.minor = element_blank(),
        panel.background = element_rect(fill="gray96"),
        plot.background  = element_blank(),
        axis.text.x = element_text( colour = 'black' ,size = 7),
        axis.text.y = element_text( colour = 'black',size = 7),
        axis.title= element_text( colour = 'black' ,size = 7, face="italic"),
        axis.ticks = element_line(size=0.1),
        panel.spacing = unit(1, "mm"),
        legend.position="none")
plots[[i]] <- plot

}
multiplot(plotlist = plots, cols = 5,layout = matrix(1:20, nrow=4, byrow = TRUE))
```

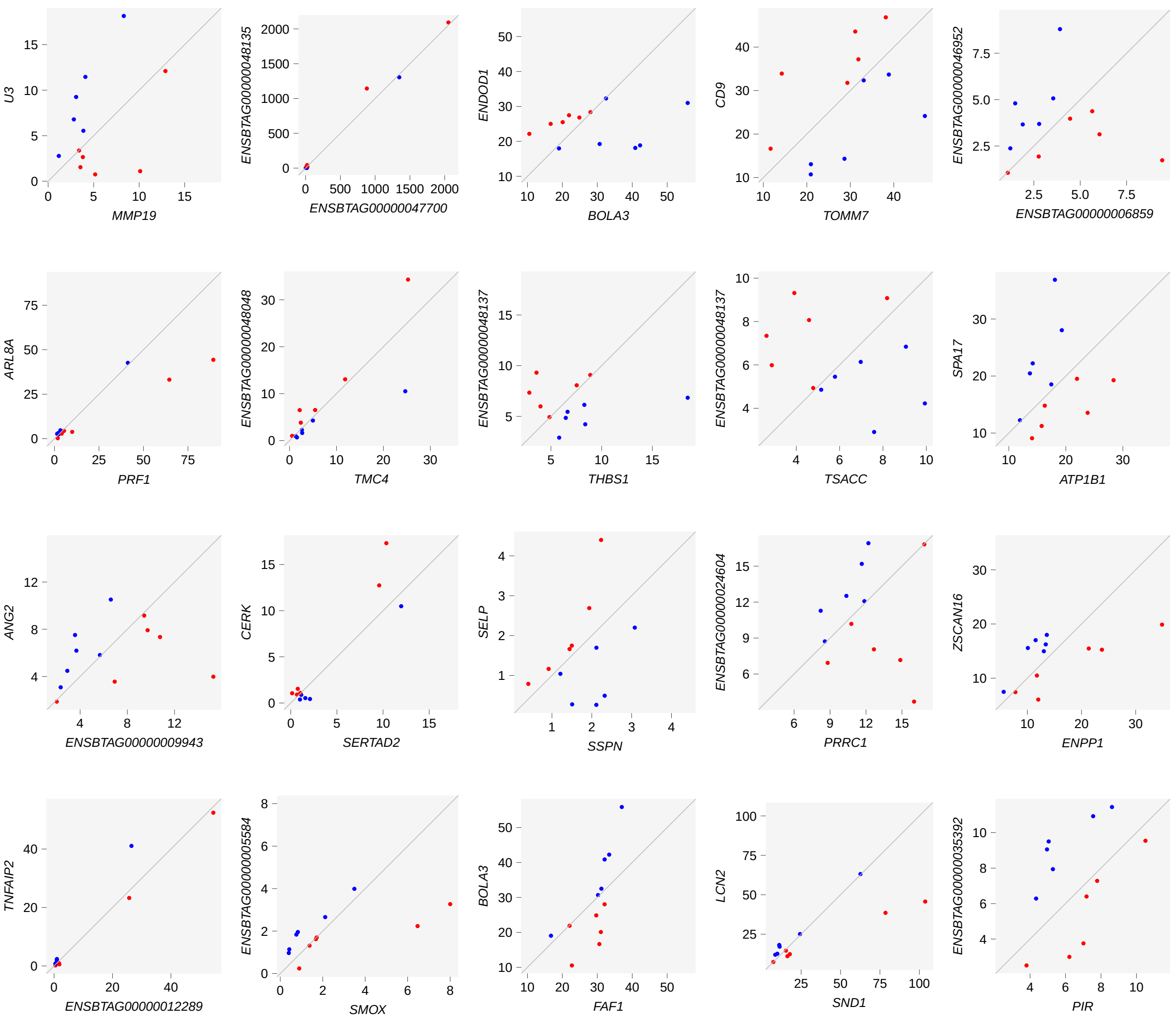

Fig 3d

First, we calculated the clusters, and the certainty of their formation, using the ratio of gene expression for the top 20 TSPs.

```
data_for_TSP_chart<-data.frame()
data_for_TSP_chart_multiple<-data.frame()
for (i in c(1:20)) {
  data_for_TSP_chart<-FPKM_stationB[c(tsp_stationB_index[i,1],tsp_stationB_index[i,2]),]
  gene_1<-rownames(data_for_TSP_chart)[1]
  gene_2<-rownames(data_for_TSP_chart)[2]
  data_for_TSP_chart<- data.frame(t( data_for_TSP_chart ))
  data_for_TSP_chart$group<-factor(group, levels=c("preg_AI", "not_preg"))
  data_for_TSP_chart$samples<-rownames(data_for_TSP_chart)
  data_for_TSP_chart<-data_for_TSP_chart[with(data_for_TSP_chart, order(group,samples)), ]

  if(data_for_TSP_chart[1,1] > data_for_TSP_chart[1,2]) {

    data_for_TSP_chart$ratio<-log2(data_for_TSP_chart[,1]+1) - log2(data_for_TSP_chart[,2]+1)
```

```
    data_for_TSP_chart$gene_1<-gene_1
    data_for_TSP_chart$gene_2<-gene_2

  } else{data_for_TSP_chart$ratio<-log2(data_for_TSP_chart[,2]+1) - log2(data_for_TSP_chart[,1]+1)
  data_for_TSP_chart$gene_1<-gene_2
  data_for_TSP_chart$gene_2<-gene_1
  }

  colnames(data_for_TSP_chart)<-c("gene1", "gene2", "group", "samples", "ratio", "gene_1", "gene_2" )
  data_for_TSP_chart_multiple<-rbind(data_for_TSP_chart_multiple,data_for_TSP_chart)
}
head(data_for_TSP_chart_multiple)


data_for_TSP_heatmap <- dcast(data_for_TSP_chart_multiple,
                              formula = gene_1 + gene_2 ~ samples, value.var = "ratio")
head(data_for_TSP_heatmap)
dim(data_for_TSP_heatmap)
data_for_TSP_heatmap$gene_1_symbol<-
  annotation_ensembl_biomart$external_gene_name[match(data_for_TSP_heatmap$gene_1,
                                                      annotation_ensembl_biomart$ensembl_gene_id)]
data_for_TSP_heatmap$gene_2_symbol<-
  annotation_ensembl_biomart$external_gene_name[match(data_for_TSP_heatmap$gene_2,
                                                      annotation_ensembl_biomart$ensembl_gene_id)]
data_for_TSP_heatmap$gene_1_symbol<-
  ifelse(!(data_for_TSP_heatmap$gene_1_symbol==""), data_for_TSP_heatmap$gene_1_symbol,
         data_for_TSP_heatmap$gene_1)
data_for_TSP_heatmap$gene_2_symbol<-
  ifelse(!(data_for_TSP_heatmap$gene_2_symbol==""), data_for_TSP_heatmap$gene_2_symbol,
         data_for_TSP_heatmap$gene_2)
data_for_TSP_heatmap$gene_order<-paste(data_for_TSP_heatmap$gene_2_symbol, ">",
                                       data_for_TSP_heatmap$gene_1_symbol, sep=" ")


result<-pvclust(data_for_TSP_heatmap[,c(3:14)], method.dist="euclidean",
                method.hclust="average",nboot=5000, parallel=FALSE)
plot(result)
```

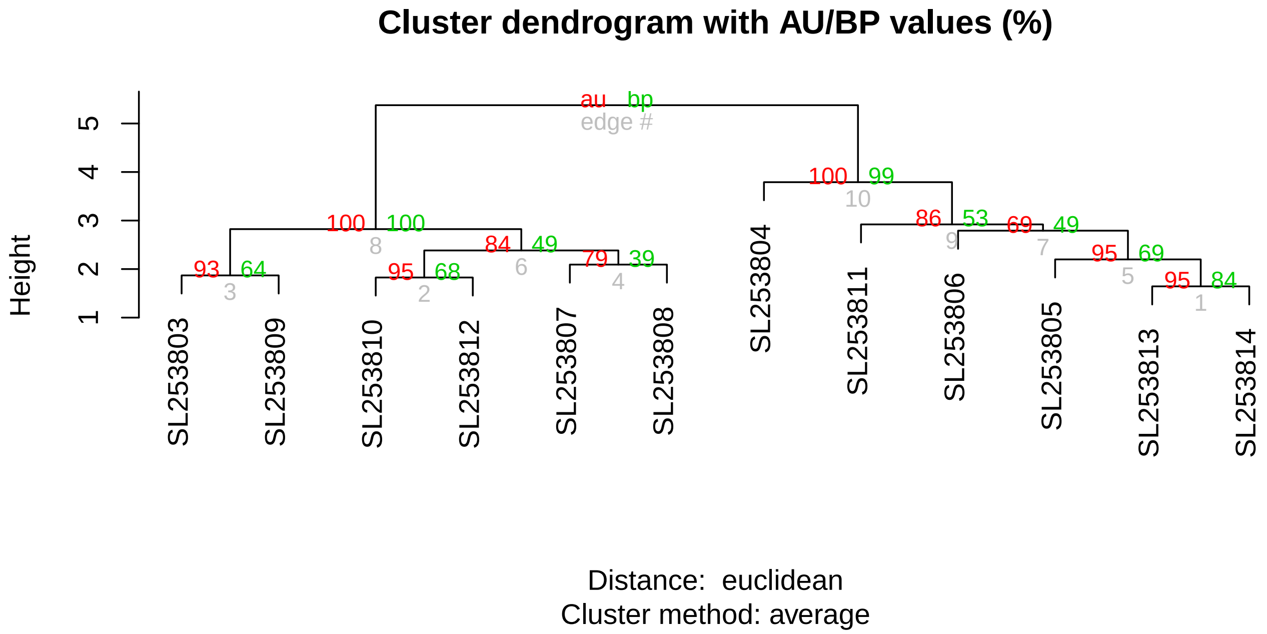

Next we plotted the heatmap.

```
row_annotation<-data_for_TSP_heatmap$gene_order
row_annotation<-row_annotation[hclust(dist(data_for_TSP_heatmap[,c(3:14)],
                                          method = "manhattan"),method = "complete")$order]
draw(
Heatmap(data_for_TSP_heatmap[,c(3:14)],
        name = "Log2(Gene1)-Log2(Gene2)",
        cluster_rows= hclust(dist(data_for_TSP_heatmap[,c(3:14)],
                                  method = "manhattan"),method = "complete"),
        cluster_columns= color_branches( result$hclust , k=2, col=c("blue","red")),
        show_column_names = TRUE,
        show_row_dend = FALSE,
        col = colorRamp2(c(-3, 0, 3), c("red", "white", "blue")),
        heatmap_legend_param = list(color_bar = "continuous",
                                    legend_direction = "horizontal",
                                    title_position = "lefttop",grid_height = unit(2, "mm"),
                                    gap=unit(1,"mm"),labels_gp = gpar(fontsize = 7),
                                    title_gp = gpar(fontsize = 7))) +

        rowAnnotation(link = row_anno_link(at= hclust(dist(data_for_TSP_heatmap[,c(3:14)],
                                          method = "manhattan"),method = "complete")$order,
                                          labels = row_annotation,
                                          labels_gp = gpar(fontsize = 8) ),
                      width = unit(2, "mm") +
        max_text_width(row_annotation, gp = gpar(fontsize = 8)))
        ,heatmap_legend_side = "bottom"
)
```

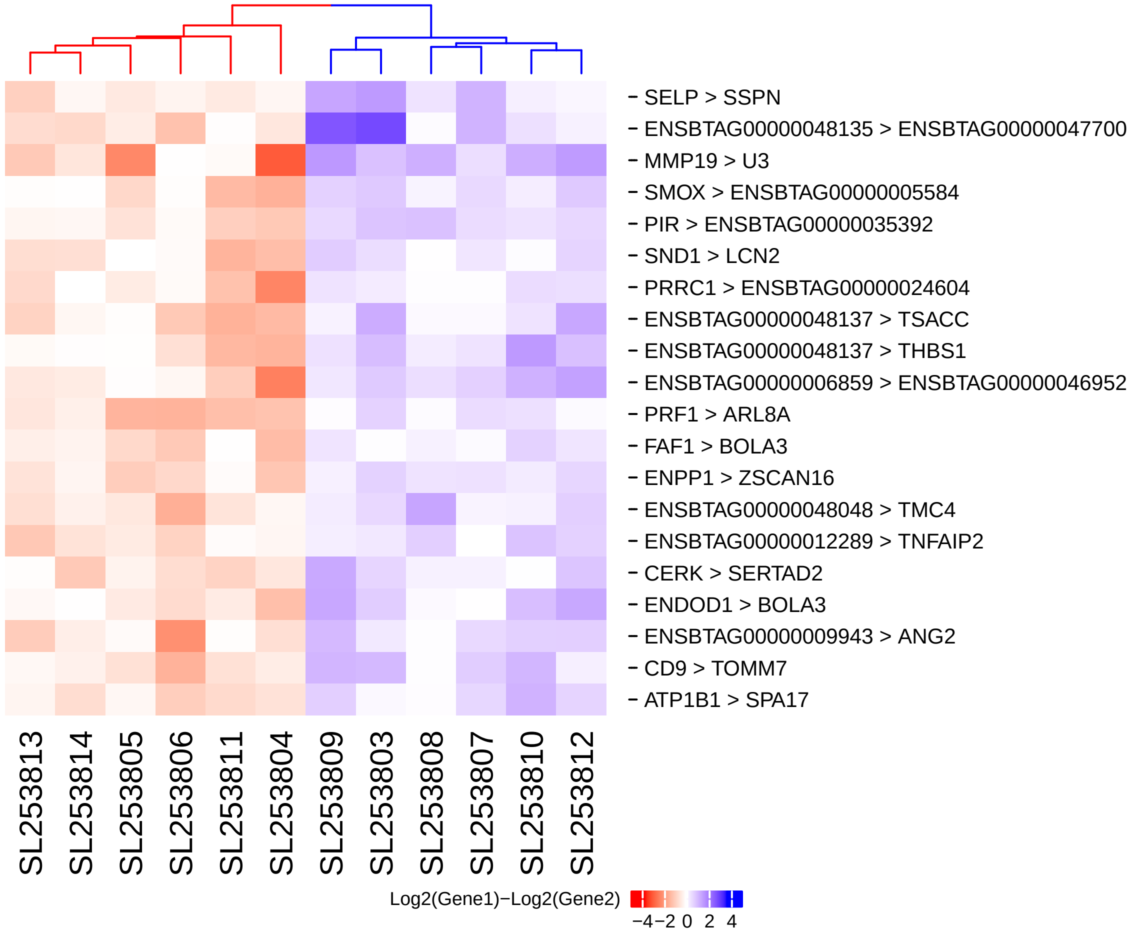

Fig 3e

Next we plot the scatterplots for the 2 TSPs for all 23 samples.

```
tsp_stationAandB_index<-as.data.frame(tsp_stationAandB$index)
tsp_stationAandB_index[,3]<-tsp_stationAandB$score
tsp_stationAandB_index[,4]<-tsp_stationAandB$tspscore
tsp_stationAandB_index<-tsp_stationAandB_index[with(tsp_stationAandB_index, order(-V3)), ]
tsp_stationAandB_index<-as.data.frame(tsp_stationAandB_index)

group_a<-c("preg_AI","preg_AI","preg_AI","preg_nb","preg_nb","preg_nb"
           ,"preg_AI","preg_AI","preg_nb","preg_nb","preg_AI",
         "preg_AI","not_preg","not_preg","not_preg","preg_AI",
         "preg_AI","preg_AI","preg_AI","not_preg","preg_AI",
         "not_preg","not_preg")

data_for_TSP_chart<-data.frame()
data_for_TSP_chart_multiple<-data.frame()
for (i in c(1:2)) {
  data_for_TSP_chart<-FPKM_stationAandB[c(tsp_stationAandB_index[i,1],tsp_stationAandB_index[i,2]),]
  gene_1<-rownames(data_for_TSP_chart)[1]
  gene_2<-rownames(data_for_TSP_chart)[2]
  data_for_TSP_chart<- data.frame(t( data_for_TSP_chart ))
```

```
  data_for_TSP_chart$group<-factor(group_a, levels=c("preg_AI","not_preg","preg_nb"))
  data_for_TSP_chart$chart<-i
  data_for_TSP_chart$gene_1<-gene_1
  data_for_TSP_chart$gene_2<-gene_2
  colnames(data_for_TSP_chart)<-c("gene_1_fpkm","gene_2_fpkm","group","chart","gene_1","gene_2")
  data_for_TSP_chart_multiple<-rbind(data_for_TSP_chart_multiple,data_for_TSP_chart)
}

data_for_TSP_chart_multiple$gene_1_symbol<-
  annotation_ensembl_biomart$external_gene_name[match(data_for_TSP_chart_multiple$gene_1,
                                                       annotation_ensembl_biomart$ensembl_gene_id)]
data_for_TSP_chart_multiple$gene_2_symbol<-
  annotation_ensembl_biomart$external_gene_name[match(data_for_TSP_chart_multiple$gene_2,
                                                       annotation_ensembl_biomart$ensembl_gene_id)]

data_for_TSP_chart_multiple$gene_1_symbol<-
  ifelse(!(data_for_TSP_chart_multiple$gene_1_symbol==""), data_for_TSP_chart_multiple$gene_1_symbol,
         data_for_TSP_chart_multiple$gene_1)
data_for_TSP_chart_multiple$gene_2_symbol<-
  ifelse(!(data_for_TSP_chart_multiple$gene_2_symbol==""), data_for_TSP_chart_multiple$gene_2_symbol,
         data_for_TSP_chart_multiple$gene_2)
```

```
plots <- list()
for (i  in c(1:2)){
data_for_TSP_chart_multiple_a<-data_for_TSP_chart_multiple[data_for_TSP_chart_multiple$chart %in% i,]
plot<-ggplot(data=data_for_TSP_chart_multiple_a, aes(x=gene_1_fpkm,y=gene_2_fpkm))+
  geom_point(aes(colour=group),size=1)+
  scale_color_manual(values=c("blue","red", "green"))+
  geom_abline(intercept = 0, slope = 1, color="gray",size=0.2)+
  scale_y_continuous(name=data_for_TSP_chart_multiple_a$gene_2_symbol[1] ,
                     limits = c(min(data_for_TSP_chart_multiple_a$gene_1_fpkm,
                                    data_for_TSP_chart_multiple_a$gene_2_fpkm),
                                max(data_for_TSP_chart_multiple_a$gene_1_fpkm,
                                    data_for_TSP_chart_multiple_a$gene_2_fpkm)))+
  scale_x_continuous(name=data_for_TSP_chart_multiple_a$gene_1_symbol[1],
                     limits = c(min(data_for_TSP_chart_multiple_a$gene_1_fpkm,
                                    data_for_TSP_chart_multiple_a$gene_2_fpkm),
                                max(data_for_TSP_chart_multiple_a$gene_1_fpkm,
                                    data_for_TSP_chart_multiple_a$gene_2_fpkm)))+
  #facet_wrap( ~ chart + gene_1_symbol + gene_2_symbol, nrow=2)+
  theme(aspect.ratio = 1,
        panel.grid.major = element_blank(),
        panel.grid.minor = element_blank(),
        panel.background = element_rect(fill="gray96"),
        plot.background  = element_blank(),
        axis.text.x = element_text( colour = 'black' ,size = 10),
        axis.text.y = element_text( colour = 'black',size = 10),
        axis.title= element_text( colour = 'black' ,size = 10, face="italic"),
        axis.ticks = element_line(size=0.1),
        panel.spacing = unit(1, "mm"),
        legend.position="none")
plots[[i]] <- plot

}
```

```r
multiplot(plotlist = plots, cols = 2,layout = matrix(1:2, nrow=1, byrow = TRUE))
```

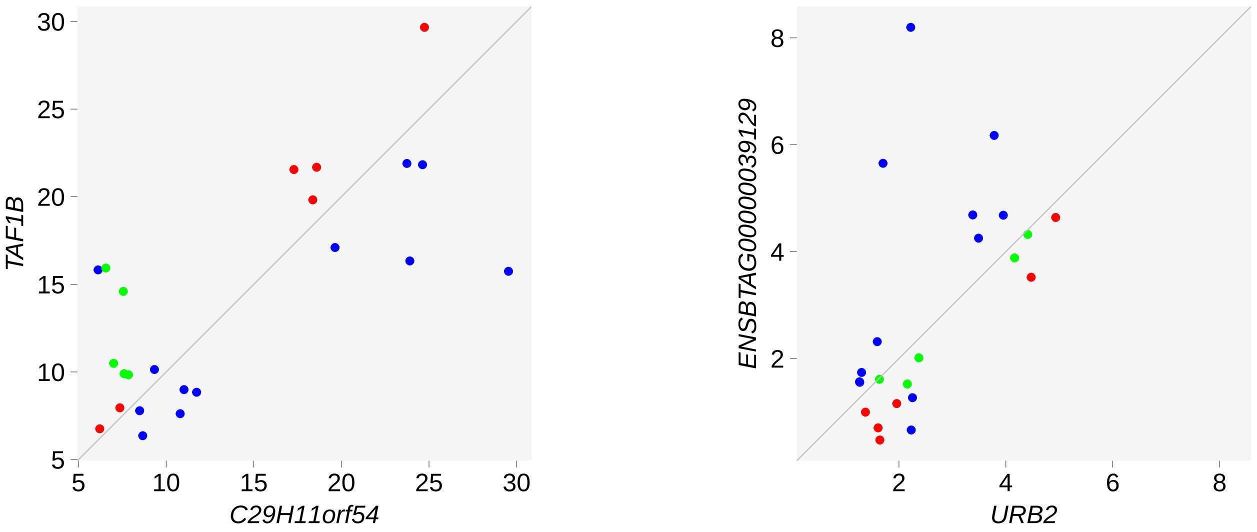

Next we calculate the probability of significance of the separation of 10 out of 12 heifers pregnant by AI according to TSP pair

```r
phyper(10-1, 12, 11, 12, lower.tail = FALSE, log.p = FALSE)
```

```
## [1] 0.002783123
```

```r
devtools::session_info()
```

```
## Session info -------------------------------------------------------------

##  setting  value
##  version  R version 3.4.3 (2017-11-30)
##  system   x86_64, darwin15.6.0
##  ui       X11
##  language (EN)
##  collate  en_US.UTF-8
##  tz       America/Chicago
##  date     2017-12-14

## Packages -----------------------------------------------------------------

##  package              * version  date
##  acepack                1.4.1    2016-10-29
##  annotate             * 1.54.0   2017-04-25
##  AnnotationDbi        * 1.38.2   2017-07-27
##  assertthat             0.2.0    2017-04-11
##  backports              1.1.1    2017-09-25
##  base                 * 3.4.3    2017-12-07
##  base64enc              0.1-3    2015-07-28
##  bigmemory            * 4.5.31   2017-11-20
##  bigmemory.sri        * 0.1.3    2014-08-18
##  bindr                  0.1      2016-11-13
##  bindrcpp               0.2      2017-06-17
##  Biobase              * 2.36.2   2017-05-04
##  BiocGenerics         * 0.22.1   2017-10-07
##  BiocParallel           1.10.1   2017-05-03
##  biomaRt              * 2.35.4   2017-12-14
```

```
##  bit                    1.1-12   2014-04-09
##  bit64                  0.9-7    2017-05-08
##  bitops                 1.0-6    2013-08-17
##  blob                   1.1.0    2017-06-17
##  caTools                1.17.1   2014-09-10
##  checkmate              1.8.5    2017-10-24
##  circlize             * 0.4.2    2017-11-18
##  class                  7.3-14   2015-08-30
##  cluster                2.0.6    2017-03-10
##  codetools              0.2-15   2016-10-05
##  colorspace             1.3-2    2016-12-14
##  compiler               3.4.3    2017-12-07
##  ComplexHeatmap       * 1.14.0   2017-02-15
##  curl                   3.1      2017-12-12
##  data.table             1.10.4-3 2017-10-27
##  datasets             * 3.4.3    2017-12-07
##  DBI                    0.7      2017-06-18
##  DelayedArray         * 0.2.7    2017-06-03
##  dendextend           * 1.6.0    2017-11-13
##  DEoptimR               1.0-8    2016-11-19
##  DESeq2               * 1.16.1   2017-05-06
##  devtools               1.13.4   2017-11-09
##  digest                 0.6.12   2017-01-27
##  diptest                0.75-7   2016-12-05
##  doParallel           * 1.0.11   2017-09-28
##  dplyr                  0.7.4    2017-09-28
##  edgeR                * 3.18.1   2017-05-06
##  evaluate               0.10.1   2017-06-24
##  flexmix                2.3-14   2017-04-28
##  foreach              * 1.4.4    2017-12-12
##  foreign                0.8-69   2017-06-22
##  Formula                1.2-2    2017-07-10
##  fpc                    2.1-10   2015-08-14
##  futile.logger        * 1.4.3    2016-07-10
##  futile.options         1.0.0    2010-04-06
##  gdata                  2.18.0   2017-06-06
##  genefilter             1.58.1   2017-05-06
##  geneplotter            1.54.0   2017-04-25
##  GenomeInfoDb         * 1.12.3   2017-10-05
##  GenomeInfoDbData       0.99.0   2017-06-03
##  GenomicRanges        * 1.28.6   2017-10-04
##  GetoptLong             0.1.6    2017-03-07
##  ggplot2              * 2.2.1    2016-12-30
##  GlobalOptions          0.0.12   2017-05-21
##  glue                   1.2.0    2017-10-29
##  gplots               * 3.0.1    2016-03-30
##  graphics             * 3.4.3    2017-12-07
##  grDevices            * 3.4.3    2017-12-07
##  grid                 * 3.4.3    2017-12-07
##  gridExtra            * 2.3      2017-09-09
##  gtable                 0.2.0    2016-02-26
##  gtools               * 3.5.0    2015-05-29
##  Heatplus             * 2.22.0   2017-04-25
##  highr                  0.6      2016-05-09
```

```
##  Hmisc                  4.0-3    2017-05-02
##  htmlTable              1.11.0   2017-12-01
##  htmltools              0.3.6    2017-04-28
##  htmlwidgets            0.9      2017-07-10
##  httr                   1.3.1    2017-08-20
##  IRanges              * 2.10.5   2017-10-08
##  iterators            * 1.0.9    2017-12-12
##  kernlab                0.9-25   2016-10-03
##  KernSmooth             2.23-15  2015-06-29
##  knitr                * 1.17     2017-08-10
##  labeling               0.3      2014-08-23
##  lambda.r               1.2      2017-09-16
##  lattice                0.20-35  2017-03-25
##  latticeExtra           0.6-28   2016-02-09
##  lazyeval               0.2.1    2017-10-29
##  limma                * 3.32.10  2017-10-13
##  locfit                 1.5-9.1  2013-04-20
##  magrittr               1.5      2014-11-22
##  MASS                   7.3-47   2017-02-26
##  Matrix                 1.2-12   2017-11-20
##  matrixStats          * 0.52.2   2017-04-14
##  mclust                 5.4      2017-11-22
##  memoise                1.1.0    2017-04-21
##  methods              * 3.4.3    2017-12-07
##  modeltools             0.2-21   2013-09-02
##  munsell                0.4.3    2016-02-13
##  mvtnorm                1.0-6    2017-03-02
##  nnet                   7.3-12   2016-02-02
##  org.Bt.eg.db         * 3.4.1    2017-06-03
##  parallel             * 3.4.3    2017-12-07
##  pkgconfig              2.0.1    2017-03-21
##  plyr                   1.8.4    2016-06-08
##  prabclus               2.2-6    2015-01-14
##  prettyunits            1.0.2    2015-07-13
##  progress               1.1.2    2016-12-14
##  purrr                  0.2.4    2017-10-18
##  pvclust              * 2.0-0    2015-10-23
##  R6                     2.2.2    2017-06-17
##  RColorBrewer         * 1.1-2    2014-12-07
##  Rcpp                   0.12.14  2017-11-23
##  RCurl                  1.95-4.8 2016-03-01
##  reshape2             * 1.4.3    2017-12-11
##  rjson                  0.2.15   2014-11-03
##  rlang                  0.1.4    2017-11-05
##  rmarkdown              1.8      2017-11-17
##  robustbase             0.92-8   2017-11-01
##  rpart                  4.1-11   2017-03-13
##  rprojroot              1.2      2017-01-16
##  RSQLite                2.0      2017-06-19
##  rstudioapi             0.7      2017-09-07
##  S4Vectors            * 0.14.7   2017-10-08
##  scales                 0.5.0    2017-08-24
##  shape                  1.4.3    2017-08-16
##  splines                3.4.3    2017-12-07
```

```
##  statmod                1.4.30   2017-06-18
##  stats                * 3.4.3    2017-12-07
##  stats4               * 3.4.3    2017-12-07
##  stringi                1.1.6    2017-11-17
##  stringr                1.2.0    2017-02-18
##  SummarizedExperiment * 1.6.5    2017-09-29
##  survival               2.41-3   2017-04-04
##  tibble                 1.3.4    2017-08-22
##  tidyr                  0.7.2    2017-10-16
##  tools                  3.4.3    2017-12-07
##  trimcluster            0.1-2    2012-10-29
##  tspair               * 1.34.0   2017-04-25
##  utils                * 3.4.3    2017-12-07
##  VennDiagram          * 1.6.18   2017-11-21
##  viridis                0.4.0    2017-03-27
##  viridisLite            0.2.0    2017-03-24
##  whisker                0.3-2    2013-04-28
##  withr                  2.1.0    2017-11-01
##  XML                  * 3.98-1.9 2017-06-19
##  xtable                 1.8-2    2016-02-05
##  XVector                0.16.0   2017-04-25
##  yaml                   2.1.16   2017-12-12
##  zlibbioc               1.22.0   2017-04-25
##  source
##  CRAN (R 3.4.0)
##  Bioconductor
##  Bioconductor
##  cran (@0.2.0)
##  CRAN (R 3.4.2)
##  local
##  CRAN (R 3.4.0)
##  CRAN (R 3.4.3)
##  CRAN (R 3.4.0)
##  CRAN (R 3.4.0)
##  CRAN (R 3.4.0)
##  Bioconductor
##  Bioconductor
##  Bioconductor
##  Github (grimbough/biomaRt@de7af3a)
##  CRAN (R 3.4.0)
##  CRAN (R 3.4.0)
##  CRAN (R 3.4.0)
##  CRAN (R 3.4.0)
##  CRAN (R 3.4.0)
##  CRAN (R 3.4.2)
##  CRAN (R 3.4.2)
##  CRAN (R 3.4.3)
##  CRAN (R 3.4.3)
##  CRAN (R 3.4.3)
##  CRAN (R 3.4.0)
##  local
##  Bioconductor (R 3.4.0)
##  CRAN (R 3.4.3)
##  CRAN (R 3.4.2)
```

```
##   local
##   CRAN (R 3.4.0)
##   Bioconductor
##   CRAN (R 3.4.2)
##   CRAN (R 3.4.0)
##   Bioconductor
##   CRAN (R 3.4.2)
##   CRAN (R 3.4.0)
##   CRAN (R 3.4.0)
##   CRAN (R 3.4.2)
##   CRAN (R 3.4.2)
##   Bioconductor
##   CRAN (R 3.4.0)
##   CRAN (R 3.4.0)
##   CRAN (R 3.4.3)
##   CRAN (R 3.4.3)
##   CRAN (R 3.4.1)
##   CRAN (R 3.4.0)
##   CRAN (R 3.4.0)
##   CRAN (R 3.4.0)
##   CRAN (R 3.4.0)
##   Bioconductor
##   Bioconductor
##   Bioconductor
##   Bioconductor
##   Bioconductor
##   CRAN (R 3.4.0)
##   CRAN (R 3.4.0)
##   CRAN (R 3.4.0)
##   CRAN (R 3.4.2)
##   CRAN (R 3.4.0)
##   local
##   local
##   local
##   CRAN (R 3.4.1)
##   CRAN (R 3.4.0)
##   CRAN (R 3.4.0)
##   Bioconductor
##   CRAN (R 3.4.0)
##   CRAN (R 3.4.0)
##   CRAN (R 3.4.3)
##   CRAN (R 3.4.0)
##   CRAN (R 3.4.0)
##   CRAN (R 3.4.1)
##   Bioconductor
##   CRAN (R 3.4.3)
##   CRAN (R 3.4.0)
##   CRAN (R 3.4.3)
##   CRAN (R 3.4.1)
##   CRAN (R 3.4.0)
##   CRAN (R 3.4.1)
##   CRAN (R 3.4.3)
##   CRAN (R 3.4.0)
##   CRAN (R 3.4.2)
```

```
##  Bioconductor
##  CRAN (R 3.4.0)
##  CRAN (R 3.4.0)
##  CRAN (R 3.4.3)
##  CRAN (R 3.4.3)
##  CRAN (R 3.4.0)
##  CRAN (R 3.4.3)
##  CRAN (R 3.4.0)
##  local
##  CRAN (R 3.4.0)
##  CRAN (R 3.4.0)
##  CRAN (R 3.4.0)
##  CRAN (R 3.4.3)
##  Bioconductor
##  local
##  CRAN (R 3.4.0)
##  CRAN (R 3.4.0)
##  CRAN (R 3.4.0)
##  cran (@1.0.2)
##  cran (@1.1.2)
##  CRAN (R 3.4.2)
##  CRAN (R 3.4.0)
##  CRAN (R 3.4.0)
##  CRAN (R 3.4.0)
##  CRAN (R 3.4.3)
##  CRAN (R 3.4.0)
##  CRAN (R 3.4.3)
##  CRAN (R 3.4.0)
##  CRAN (R 3.4.2)
##  CRAN (R 3.4.2)
##  CRAN (R 3.4.2)
##  CRAN (R 3.4.3)
##  CRAN (R 3.4.0)
##  CRAN (R 3.4.0)
##  CRAN (R 3.4.1)
##  Bioconductor
##  CRAN (R 3.4.1)
##  CRAN (R 3.4.1)
##  local
##  CRAN (R 3.4.0)
##  local
##  local
##  CRAN (R 3.4.2)
##  CRAN (R 3.4.0)
##  Bioconductor
##  CRAN (R 3.4.3)
##  CRAN (R 3.4.1)
##  CRAN (R 3.4.2)
##  local
##  CRAN (R 3.4.0)
##  Bioconductor
##  local
##  CRAN (R 3.4.1)
##  CRAN (R 3.4.0)
```

```
## CRAN (R 3.4.0)
## CRAN (R 3.4.0)
## CRAN (R 3.4.2)
## CRAN (R 3.4.1)
## CRAN (R 3.4.0)
## Bioconductor
## CRAN (R 3.4.3)
## Bioconductor
```
